# Predicting response to GLP-1–based therapy in MASLD/MASH: an evidence-tiered, endpoint-specific framework built on host genotype and gut microbial features

**DOI:** 10.3389/fendo.2026.1962068

**Published:** 2026-10-27

**Authors:** Guojian Zhu, Yucao Huang, Yun Huang, Wenlong Yang

**Affiliations:** Department of Infectious Diseases, The Second Affiliated Hospital, Jiangxi Medical College, Nanchang University, Nanchang, Jiangxi, China

**Keywords:** genetic risk score, GLP-1 receptor agonist, gut microbiome, metabolic dysfunction-associated steatohepatitis, PNPLA3, response heterogeneity, semaglutide

## Abstract

Incretin-based therapy is now central to treating metabolic dysfunction-associated steatohepatitis (MASH), but the benefit is uneven. In the ESSENCE trial, semaglutide resolved steatohepatitis without worsening fibrosis in 62.9% of patients; placebo did so in 34.3%. No one can yet tell in advance which patient will respond. Weight loss explains most of the difference between trials, but it cannot be prescribed. This review asks whether biology measured before the first dose can predict who will benefit. Hepatocytes carry almost no GLP-1 receptor, so the liver benefit arrives indirectly, through weight loss and through gut–liver signaling. Both routes can be influenced from outside the liver, which points to two measurable domains: host genotype, fixed and assayable at baseline, and gut microbial features. For a dietary prebiotic, baseline microbial features have already forecast response, with an externally validated area under the curve of 0.74 (95% CI 0.66–0.81). Nothing equivalent has been tested for a GLP-1 receptor agonist. The genotype evidence comes from dietary weight loss rather than from a drug. Both pillars therefore rest on interventions other than incretin therapy, and the framework grades them accordingly. We assemble this evidence into a framework with two rules. Findings are graded by how mature the evidence behind them is. Liver and cardiovascular benefits are predicted separately, because the two do not track together. The output is a probability rather than a label. One conclusion is already usable: a predicted poor liver response is not, on its own, a reason to withhold or stop a GLP-1 receptor agonist.

## 1 Introduction

Metabolic dysfunction-associated steatotic liver disease (MASLD) has become the most common chronic liver disease worldwide, affecting an estimated 38% of adults. Its prevalence continues to rise in parallel with obesity and type 2 diabetes (1). The 2023 multisociety Delphi consensus replaced the term non-alcoholic fatty liver disease with MASLD. It was intended to remove stigma and to anchor the diagnosis to cardiometabolic dysfunction, which sharpened how the disease spectrum is defined and studied (2). Within this spectrum, the subset of patients with metabolic dysfunction-associated steatohepatitis (MASH) and progressive fibrosis carries the greatest risk of cirrhosis, hepatocellular carcinoma, and liver-related death. The stage of fibrosis remains the dominant determinant of long-term hepatic outcomes (3, 4). Identifying and treating this at-risk population has therefore become the central objective of contemporary hepatology.

For most of its history, MASH lacked any approved pharmacological therapy, and management rested almost entirely on weight loss through lifestyle modification. This changed within a remarkably short interval. In 2024, resmetirom, a liver-directed thyroid hormone receptor-β agonist, became the first drug approved for MASH with fibrosis. The phase 3 MAESTRO-NASH trial had demonstrated both steatohepatitis resolution and fibrosis improvement (5). The incretin-based agents followed closely. The phase 2 SYNERGY-NASH trial showed that the dual GIP/GLP-1 receptor agonist tirzepatide resolved MASH in a dose-dependent manner (6). The phase 3 ESSENCE trial then led to the approval of semaglutide for MASH with moderate to advanced fibrosis (7, 8). Within roughly 18 months, the field moved from having no disease-specific therapy to possessing several, and metabolic pharmacotherapy is now positioned at the center of MASH management.

Yet, the same pivotal trials expose a problem that will define the next phase of care: response is heterogeneous. In ESSENCE, 62.9% of semaglutide-treated patients achieved resolution of steatohepatitis without worsening of fibrosis, but so did 34.3% of those receiving placebo. A further 37.1% of treated patients did not meet the endpoint, a categorical failure that does not imply the absence of any biological effect (7). At present, clinicians have no validated way to predict, before initiating treatment, which patient will respond. That omission is increasingly untenable as therapeutic options, costs, and gastrointestinal side effects all grow.

Two lines of evidence suggest that this heterogeneity is not random but is written, at least in part, into host biology. The first is genetic. Common variants in PNPLA3, TM6SF2, MBOAT7, and HSD17B13 shape susceptibility to steatosis and the risk of progression to fibrosis. Aggregated into genetic risk scores, they also shape the probability of clinically meaningful liver events (9). Genotype also appears to modify treatment response. In carriers of the PNPLA3 risk allele, change in liver stiffness tracked weight change, although they lost weight less readily (10). These variants are stable, measurable before therapy, and biologically linked to the very pathways that metabolic drugs engage.

The second line of evidence is microbial. Hepatocytes express little or no GLP-1 receptor. The hepatic benefit of GLP-1–based therapy is therefore thought to be largely indirect, mediated through weight loss, improved insulin sensitivity, and signaling along the gut–liver axis (11). Baseline gut microbiome composition can predict response to a metabolic intervention in MASLD. This was recently demonstrated for resistant starch, where pretreatment microbial features stratified responders from non-responders with useful accuracy (12). That demonstration used a substrate delivered to the community rather than a drug acting on host receptors. The two place the microbiome on opposite sides of the response mechanism, so its transfer to incretin therapy is treated throughout as extrapolation (Section 5.3, **Table 1**). Because the microbiome is itself modifiable, it represents both a candidate biomarker and a potential therapeutic lever (8).

**Table 1:** Provenance of the load-bearing evidence: the intervention actually administered and the endpoint actually measured.

#	Finding as used in this review	Ref	Intervention actually administered	Endpoint actually measured	Tier
1	Semaglutide resolves steatohepatitis in 62.9% versus 34.3% on placebo	7,8	Semaglutide 2.4 mg	Histology, paired biopsy	1
2	Tirzepatide resolves MASH dose-dependently	6	Tirzepatide, dual GIP/GLP-1	Histology	1
3	Resmetirom improves steatohepatitis and fibrosis	5	**Resmetirom, a thyroid hormone receptor-β agonist**	Histology	1 for its own class only
4	Hepatic benefit of GLP-1 action is largely indirect	11,24	Mechanistic synthesis, no trial	Not applicable	Mechanism
5	Part of the lipogenic effect is weight-independent	25	Dulaglutide	Hepatic steatosis and *de novo* lipogenesis in obesity	2
6	Bile acid metabolism responds more reliably than community composition	30,31	Liraglutide and sitagliptin; metformin comparator	Microbial composition, bile acids	2
7	**Genotype modifies hepatic benefit per unit weight lost**	10	**Dietary advice, no drug administered**	**Liver stiffness, not histology**	3
8	Variant hepatocytes respond less to a directly acting hepatic drug	39	**Resmetirom *in vitro***	Cellular phenotype	3
9	**Baseline microbiota predicts response**	12	**Resistant starch, a substrate delivered to the community**	Intrahepatic triglyceride	3
10	*Prevotella* marks the better metabolic responders	50,51	**Fiber-rich and whole-grain diets**	**Body fat and weight, not a hepatic endpoint**	2, direction not stable
11	Microbial signature tracks fibrosis stage	45,46	**None; cross-sectional**	**Stage of disease, not response to therapy**	2
12	Microbial manipulation does not move hepatic endpoints	56,59-62	Synbiotic; fecal microbiota transplantation	Liver fat, validated fibrosis scores	1, negative
13	Cardiovascular benefit does not track baseline FIB-4 category	74,75	Semaglutide 2.4 mg	**MACE; hepatic phenotype not measured**	1 for the cardiovascular endpoint
14	Lifelong liver fat is not causally linked to coronary risk	79	**None; single genetic instrument**	Ischemic heart disease	2
15	Diabetes status modifies drug-specific fibrosis response	Section 6.2	**Separate trials of different agents, compared across**	**Histology, no formal interaction test**	2
16	Community composition is confounded by concomitant medication	47,48	**Metformin, not an incretin**	Composition, glucose tolerance	2

Entries in bold identify where the evidence departs from the target question, which is response of a hepatic endpoint to GLP-1–based therapy. Five of the sixteen findings administered a GLP-1 receptor agonist or dual agonist (rows 1, 2, 5, 6, and 13). The two pre-treatment pillars of the framework are rows 7 and 9, and neither of them did. This is the reason both are admitted at tier 3 under **Table 2** and may not gate a stratification decision. The table is intended to let a reader audit that mapping row by row rather than accept it.

Despite this convergence, a recent review integrating these domains aims them at risk stratification rather than response to therapy and does not examine incretin agents (13). A review of response heterogeneity concludes that evidence is insufficient for precision medicine in MASLD and that genetic factors affecting GLP-1 response have not been reported (14). To our knowledge, no prior review has integrated genotype and microbiome to predict GLP-1 response, tiered that evidence by maturity, or used the endpoint dissociation to calibrate it. This review addresses that gap, asking why phenotypically similar patients respond so differently. We synthesize the evidence linking genetic and microbial determinants to treatment response, use lean MASLD as a test case, then add the cardiovascular dimension and propose an integrated framework.

### 1.1 Literature identification

This is a narrative review, not a systematic one. Literature was identified iteratively rather than by a single prespecified search. PubMed/MEDLINE was searched throughout the writing period, to August 2026. Terms combined MASLD and MASH with GLP-1 receptor agonists and dual agonists, treatment response, the four risk loci, and the gut microbiome. Europe PMC and CrossRef supplied full texts and bibliographic records, and reference lists were screened manually.

Screening was iterative but not unstructured. A record was eligible if it reported a human or experimental study bearing on incretin response, the four risk loci, or gut microbial composition in steatotic liver disease. The primary record also had to be retrievable. A record was excluded if the hepatic outcome was neither histological nor a validated non-invasive measure. Claims resting only on a conference abstract or preprint, or effects untraceable to their source, were also excluded. Priority went to randomized trials, to histological or validated hepatic outcomes, and to negative or contested findings, which are presented alongside supportive ones.

Where small cohorts disagreed, no numerical weighting was applied. Discordance is reported as discordance. The tier assigned to a finding follows the admission criteria in **Table 2**, under which a contested result cannot rise above tier 2 however many cohorts favor it. Much of the evidence assembled here was generated under interventions other than incretin therapy. **Table 1** therefore records, for every load-bearing finding, the intervention actually administered and the endpoint actually measured. The 81 cited records span 2005 to 2026, and 55 appeared in 2020 or later. Every citation was checked against the primary record, and unverifiable claims were removed rather than softened. No protocol was registered, and no formal risk-of-bias assessment was performed. The evidence is presented in the order the argument requires. The heterogeneity to be explained comes first, then why it is modifiable from outside the liver, then the two pretreatment domains and their assembly into a framework.

**Table 2:** Admission criteria for the three evidence tiers.

Criterion	Tier 1 replicated for a hepatic endpoint	Tier 2 contested, or single-study	Tier 3 inferred by extrapolation
Study design required	≥2 independent human cohorts, or ≥1 randomized trial with within-trial testing	Human study on a relevant endpoint	Any human or experimental study whose drug class, endpoint, population, or system differs from the target question
Reproducibility required	Consistent direction of effect across sources	None; the tier admits single sources and conflicting sources	None
Analytic requirement	Formal test of the claimed effect	May rest on separate-subgroup significance without a formal interaction test	Not applicable
Endpoint	Hepatic histology, or a validated hepatic outcome	Relevant hepatic endpoint, including stage of disease	Any, including surrogate and non-hepatic
Decision function permitted	May carry weight in a stratification decision	May enter as an input carrying an explicit confidence estimate; **may not gate a decision**	May specify trial designs and generate hypotheses; **carries no weight in a stratification decision**

A reader can apply these rules to findings published after this review and place them without reference to the assignments made here. That, rather than the assignments themselves, is what the tier structure is for.

Values in bold mark the cells that carry weight in a stratification decision; entries not in bold may not gate a decision.

## 2 GLP-1–based therapies in MASLD/MASH: efficacy and the heterogeneity problem

Incretin-based agents have transformed MASH treatment, yet the trial data reveal a second, less celebrated finding. Histological response to these drugs varies widely between patients who appear clinically similar at baseline. Across the pivotal trials, only a subset of treated patients achieves resolution of steatohepatitis or improvement in fibrosis, and a meaningful fraction derives little benefit. Its magnitude and causes are the starting point for individualizing therapy, and they frame the remainder of this review (**Figure 1**).

**Figure 1:**
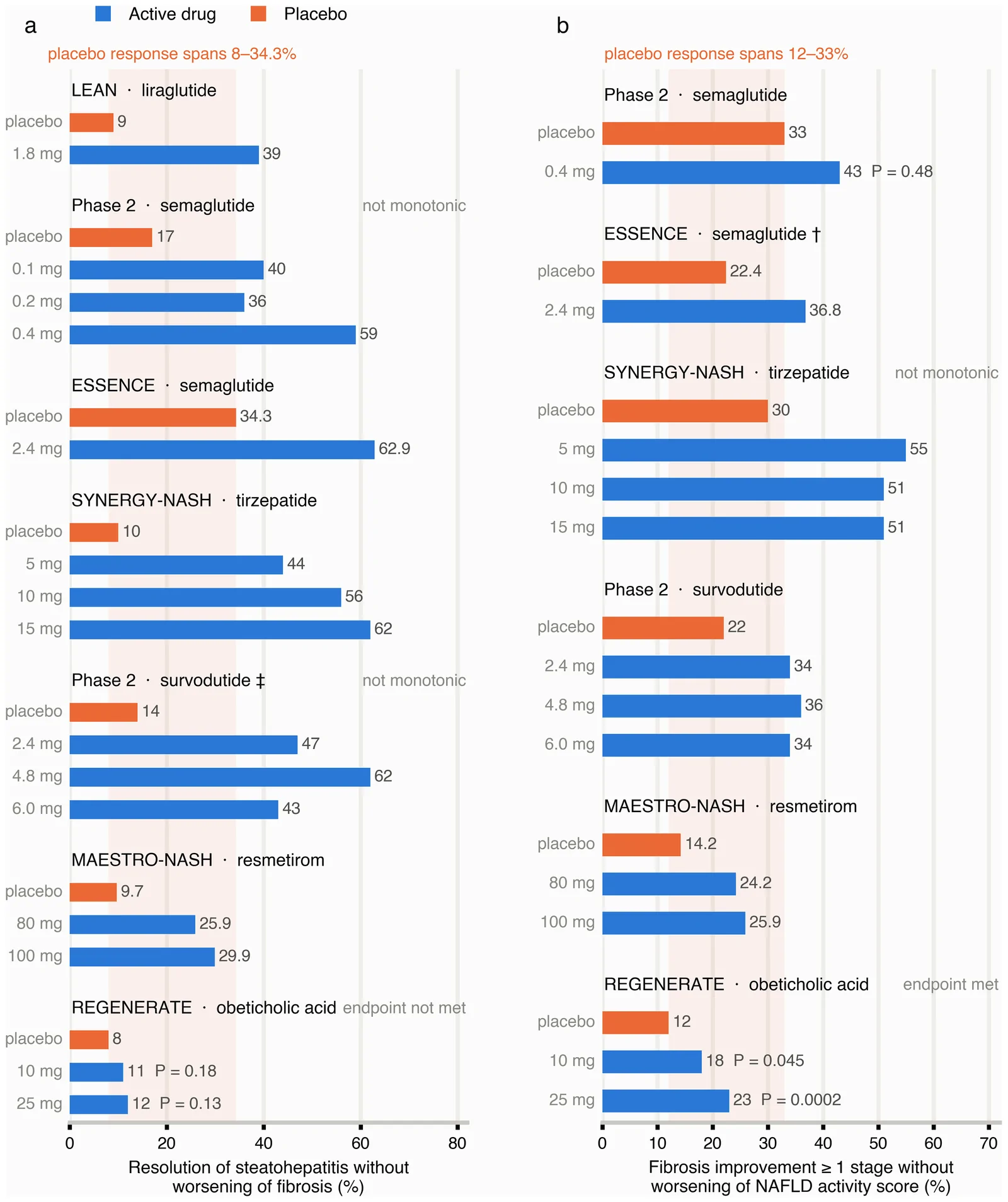
Histological response to incretin-based and comparator therapy is large in magnitude, inconsistent across doses, and set against a placebo response that varies threefold between trials. Response rates are shown as reported in each trial. They are not adjusted, pooled, or indirectly compared. Entry criteria, treatment duration, biopsy completion, and handling of missing data differ between trials. The shaded band marks the range of placebo response across trials. **(a)** Resolution of steatohepatitis without worsening of fibrosis. **(b)** Fibrosis improvement by at least one stage without worsening of the NAFLD activity score; †ESSENCE reported reduction in liver fibrosis without worsening of steatohepatitis, a differently defined endpoint. LEAN did not report fibrosis improvement and is omitted from **(b)** The dose–response relationship is not monotonic for phase 2 semaglutide in **(a)** or for tirzepatide in **(b)**.

### 2.1 Histological efficacy across the incretin trials

The clinical case for GLP-1–based therapy in MASH was built incrementally over nearly a decade. **Table 3** sets out each trial with the qualification that most limits its comparability, and three features of that record bear on prediction. The first is that efficacy is consistent in direction across four trials, three agents, and a decade. LEAN provided the first histological signal (15). Daily semaglutide confirmed it on resolution while failing to improve fibrosis stage (16). ESSENCE established both for weekly semaglutide 2.4 mg (7), and tirzepatide extended the class effect in SYNERGY-NASH (6). The second is that dose–response is not monotonic in three of these trials, which is difficult to reconcile with a simple exposure-driven mechanism. The third is that the placebo response on the resolution endpoint ranges from 8% to 34.3%, the highest value coming from the largest and most recent trial. **Table 3** gives the comparison with resmetirom, whose resolution rates in MAESTRO-NASH fall below the incretin rates (5).

**Table 3:** Histological trials of incretin-based and comparator therapy in MASH.

Trial (PMID)	Agent and class	Design and size	Population	Duration	Resolution of steatohepatitis without worsening of fibrosis	Fibrosis improvement ≥1 stage without worsening of NAFLD activity score	Qualifications bearing on comparability
LEAN (15)	Liraglutide 1.8 mg daily; GLP-1 receptor agonist	Phase 2, multicenter, 52 randomized (26: 26)	Biopsy-confirmed steatohepatitis	48 weeks	39% (9/23) vs. 9% (2/22); RR 4.3 (95% CI 1.0–17.7), P = 0.019	Not reported as improvement; fibrosis *progression* 9% vs. 36%, RR 0.2 (0.1–1.0)	Only 45 of 52 randomized patients had an end-of-treatment biopsy. Smallest trial in the table; fibrosis reported as progression, so it cannot enter a comparison of improvement rates
Phase 2 semaglutide (16)	Semaglutide 0.1, 0.2, and 0.4 mg daily; GLP-1 receptor agonist	Phase 2, 320 randomized	Biopsy-confirmed steatohepatitis, F1–F3	72 weeks	40%, 36%, 59% vs. 17%	43% (0.4 mg) vs. 33%, P = 0.48	**Not monotonic across doses.** Both the primary and the confirmatory secondary endpoints were analyzed only in the 230 of 320 patients with stage F2 or F3 fibrosis
ESSENCE (7)	Semaglutide 2.4 mg weekly; GLP-1 receptor agonist	Phase 3, 1,197 randomized 2: 1; part-1 analysis in 534 vs. 266	Biopsy-defined MASH, fibrosis stage 2 or 3	240 weeks planned; part-1 analysis at week 72	62.9% vs. 34.3%; estimated difference 28.7 percentage points (95% CI 21.1–36.2), P < 0.001	36.8% vs. 22.4%; difference 14.4 points (95% CI 7.5–21.3), P < 0.001 †	† The endpoint reported is **reduction in liver fibrosis without worsening of steatohepatitis**, which is not the endpoint used in the other rows. **Highest placebo response in the table (34.3%)**
SYNERGY-NASH (6)	Tirzepatide 5, 10, 15 mg weekly; GIP/GLP-1 receptor agonist	Phase 2, 190 randomized	Biopsy-confirmed MASH with fibrosis	52 weeks	44%, 56%, 62% vs. 10%	55%, 51%, 51% vs. 30%	**Fibrosis response not monotonic and not greatest at the highest dose.** 157 of 190 had evaluable week-52 biopsies; missing values imputed on the assumption that they would follow the pattern of the placebo group
Phase 2 survodutide (17)	Survodutide 2.4, 4.8, and 6.0 mg weekly; glucagon/GLP-1 receptor agonist	Phase 2, 1: 1: 1: 1; 293 received ≥1 dose	Biopsy-confirmed MASH, F1–F3	48 weeks (24-week escalation, 24-week maintenance)	47%, 62%, 43% vs. 14% ‡	34%, 36%, 34% vs. 22%	‡ Endpoint is **improvement in MASH** without worsening of fibrosis, not resolution. **Not monotonic: the highest dose performed worst on the primary endpoint**
MAESTRO-NASH (5)	Resmetirom 80, 100 mg daily; thyroid hormone receptor-β agonist	Phase 3, 966 in the primary analysis population	Biopsy-confirmed NASH, F1B–F3	52 weeks	25.9%, 29.9% vs. 9.7%; P < 0.001 for both	24.2%, 25.9% vs. 14.2%; P < 0.001 for both	First agent approved for MASH with fibrosis. Included here as a non-incretin comparator with a distinct mechanism
REGENERATE (18–20)	Obeticholic acid 10, 25 mg daily; FXR agonist	Phase 3, month-18 interim; 931 patients with F2–F3 in the primary analysis (311: 312: 308)	Definite NASH, NAFLD activity score ≥ 4, F2–F3 or F1 with risk factors	18-month interim	8%, 11% (P = 0.18), 12% (P = 0.13): **endpoint not met**	12%, 18% (P = 0.045), 23% (P = 0.0002): **endpoint met**	**Fibrosis improved whereas steatohepatitis resolution did not**, and resolution remained unmet on the subsequent consensus-read analysis. Dose-dependent pruritus, raised low-density lipoprotein cholesterol, cholelithiasis, and concern about drug-induced liver injury contributed to a complete response letter from the Food and Drug Administration in 2020. Never approved for MASH in any region; the licensed indication in primary biliary cholangitis is unrelated

Values are as reported by each trial and are not adjusted, pooled, or indirectly compared. Entry criteria, treatment duration, endpoint definitions, biopsy completion, and handling of missing data differ between rows, and the final column states the difference that most limits comparability in each case. Placebo response on the resolution endpoint ranges from 8% to 34.3% across the table. Three trials show a non-monotonic dose–response, and two agents separate the two endpoints in opposite directions.

Values in bold identify the comparability limitation that most constrains each trial.

Two agents outside the pure GLP-1 class bound the interpretation of these results, and both are in **Table 3**. Adding glucagon receptor agonism, as in survodutide, introduces energy expenditure and hepatic fat mobilization that GLP-1 alone does not produce. Yet, the dose–response there was best fitted by a quadratic curve (17). The FXR agonist obeticholic acid illustrates the opposite trajectory: it improved fibrosis in REGENERATE while failing to resolve steatohepatitis, the mirror image of the phase 2 semaglutide result (18, 19). Its subsequent history matters less than what its mechanism establishes. Obeticholic acid is a synthetic agonist of a host receptor. Its trial therefore shows that the host arm of bile acid signaling is druggable, not that the microbially dependent portion can be manipulated (20). One regulatory feature then constrains everything that follows. Histological response is an accelerated-approval surrogate awaiting confirmatory outcome data, and no drug for this disease has yet shown benefit on decompensation, transplantation, or death. Predicting response therefore means predicting a surrogate.

### 2.2 Variability behind the group means

These figures describe group averages that conceal substantial individual variation, and the placebo response governs how much can be inferred about any individual. With one-third of untreated patients meeting the histological endpoint, apparent response may reflect regression to the mean, lifestyle change, or sampling artifact rather than pharmacological action. Two features of the data make this variability tractable rather than noisy, and both must be stated at the level of evidence that supports them. At the study level, meta-regression across incretin and related interventions indicates that mean weight loss accounts for roughly three quarters of the between-trial heterogeneity in MASH resolution. Its share of fibrosis improvement is comparable if smaller (21). At the individual level, the evidence is coarser and shows threshold behavior. A study-level meta-analysis of six randomized trials (n = 1,726) found fibrosis benefit confined to patients achieving at least 10% weight loss (odds ratio 9.59, 95% CI 4.01 to 15.18). Below that threshold, there was no significant effect (odds ratio 1.30, 95% CI 0.92 to 1.83), a failure to demonstrate benefit rather than a demonstration of its absence (22). The study-level figure must not be read as the fraction of individual response variance attributable to weight loss. Between-trial and between-patient variation are different quantities, and conflating them is an ecological inference.

A deeper constraint applies to the whole enterprise. Parallel-group trials cannot in principle identify true individual response heterogeneity. The observed proportion of responders is fully compatible with all patients sharing a single response probability and differing only by binomial sampling. What the placebo response and the reader variability establish is that the responder label is unreliable, not that a biological non-responder phenotype exists. The object of a pretreatment framework is therefore better stated as the search for pretreatment measurable effect modifiers detectable above endpoint noise, rather than as the identification of non-responders. Designs that repeat the exposure within a patient are the only ones that identify an individual response phenotype directly. N-of-1 or crossover extensions would serve in principle and are already used for reversible outcomes in other chronic diseases. Their application to MASH is constrained rather than precluded. The histological endpoint is neither rapidly reversible nor repeatable at will, and a washout long enough to restore baseline would be hard to justify in a patient deriving benefit. A crossover extension using a continuous non-invasive measure of hepatic fat, with histology as the anchor at entry and exit, is the most feasible version. One further analysis requires no new recruitment. The individual-level dose–response relationship between weight lost and histological improvement within trials has not been published, although it is recoverable from existing participant data. It would establish whether the 10% threshold reflects a genuine inflection or an arbitrary cut point. Because hepatocytes express a negligible GLP-1 receptor, the dependence on weight loss is mechanistically expected.

Weight loss is not the only modifier, but it is the one most directly quantifiable from existing trials. Baseline metabolic status, and type 2 diabetes in particular, alters histological response in ways that differ between agents. That behavior is central to how a predictive framework must be built, so it is examined later rather than here. A further source of apparent non-response is measurement itself. Histological scoring carries substantial inter-reader variability and biopsy sampling error, which inflate placebo response rates and misclassify genuine responders as failures. Continuous artificial-intelligence–assisted quantification separates drug effect from placebo response more sensitively than categorical scoring does (23). Part of the observed heterogeneity is therefore analytical rather than biological, and distinguishing the two is a prerequisite for any credible prediction model.

### 2.3 From retrospective explanation to pretreatment prediction

Taken together, the trial evidence establishes that GLP-1–based therapies are effective for MASH at the population level while leaving individual response poorly predictable. The translational gap is specific. Weight loss accounts for most of the between-trial variation retrospectively. It cannot be prescribed or foreseen with precision at the point of care. The degree of weight loss a given patient will achieve is itself variable, and only partly under clinical control. What is missing is a set of stable, pretreatment characteristics that stratify patients before the first dose, ideally ones mechanistically tied to the pathways these drugs engage. Two candidate domains meet that requirement. Host genotype is fixed, measurable at baseline, and linked to both disease progression and drug response. The gut microbiome is dynamic yet assessable before therapy and mechanistically positioned along the indirect route through which incretin agents reach the liver. The sections that follow examine each domain in turn, beginning with the mechanistic basis for why hepatic benefit is indirect and therefore susceptible to modification by factors outside the liver.

## 3 The indirect mechanism puzzle: how GLP-1 benefits the liver

The hepatic benefit of GLP-1–based therapy poses a mechanistic paradox that bears directly on why response varies. Hepatocytes express little or no GLP-1 receptor, so these drugs cannot act on the liver as a receptor agonist reaching its target tissue (11). Their effects on steatohepatitis are relayed instead through peripheral metabolic changes and through signaling along the gut–liver axis. Because each relay is itself variable between patients, the indirectness of the mechanism is a plausible source of the heterogeneity documented in the trials (**Figure 2**).

**Figure 2:**
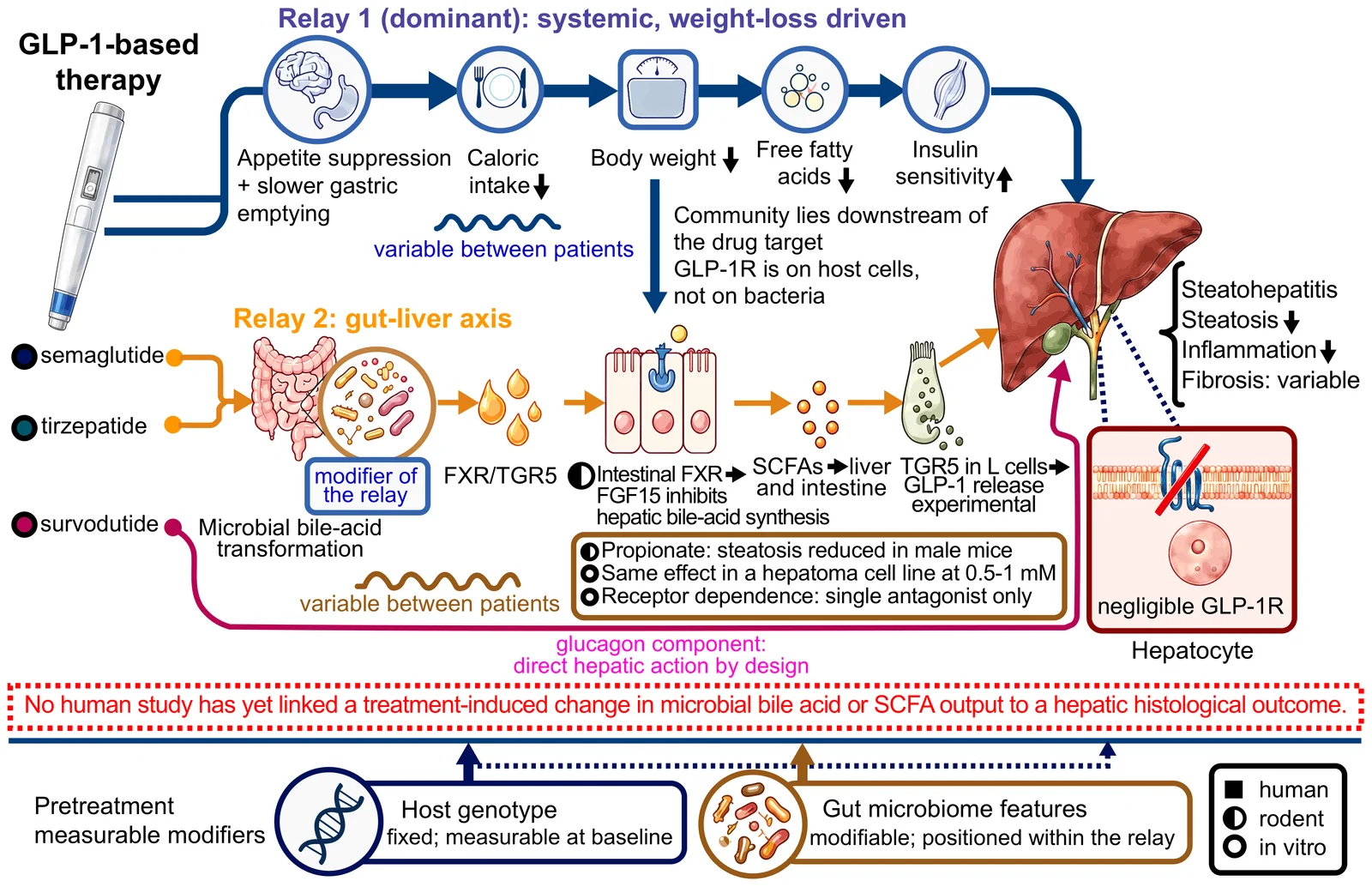
Hepatic benefit is relayed, not delivered: two routes from drug to liver, each variable between patients. Hepatocytes express little or no GLP-1 receptor, so GLP-1–based therapy cannot act on the liver as a receptor agonist reaching its target tissue. The dominant relay is systemic and driven by weight loss. Appetite suppression and slower gastric emptying reduce caloric intake and body weight, and free fatty acid delivery falls as insulin sensitivity improves. A second relay operates along the gut–liver axis. Microbial deconjugation and transformation of bile acids produce a pool that signals to liver and intestine through FXR and TGR5. Intestinal FXR activation represses hepatic bile acid synthesis through fibroblast growth factor 15, and microbially derived short-chain fatty acids act on the same tissue. The microbial community lies downstream of the drug target, since the GLP-1 receptor is expressed on host cells and not on bacteria. The community is therefore a modifier of the relay rather than an effector of the drug. The glucagon component of survodutide is the only route with a direct hepatic action by design. Symbols mark the species level at which each step rests: filled, human; half-filled, rodent; open, *in vitro*. Both relays vary between patients, which is the proposed source of the response heterogeneity shown in **Figure 1**. Host genotype and gut microbial features are the two pretreatment measurable modifiers positioned on them.

The dominant relay is systemic and weight-driven. By suppressing appetite and slowing gastric emptying, GLP-1 receptor agonists reduce caloric intake and body weight. That lowers circulating free fatty acids, improves insulin sensitivity, and diminishes the substrate supply and lipotoxic stress that sustain steatohepatitis (11, 24). This accounts for the dependence of histological response on the magnitude of weight loss noted earlier. It also accounts for the comparable reductions in hepatic steatosis achieved by matched weight loss through lifestyle intervention.

Within that relay, hepatic *de novo* lipogenesis is the step where the weight-dependent and weight-independent accounts can be told apart. Insulin resistance and hyperinsulinemia drive lipogenic transcription in the hepatocyte. Any intervention improving peripheral insulin sensitivity therefore lowers lipogenic flux without acting on the liver at all (11, 24), and improved glycemic control reduces the substrate supply to the same pathway. On this account, the lipogenic effect of an incretin is entirely downstream of weight loss, and any intervention achieving the same metabolic change would reproduce it. Experimental work complicates that reading. Dulaglutide reduced hepatic steatosis in obesity through a mechanism the authors identify as weight-independent, with reductions in *de novo* lipogenesis beyond what weight change alone would predict (25). The distinction matters for prediction, not only for mechanism. A lipogenic effect secondary to weight loss should be forecast by whatever predicts weight loss. A weight-independent component would need its own predictor, and the axes this framework assembles would not capture it. The relative weight of the two remains only partly resolved.

A second relay operates through the gut–liver axis, where the microbiome sits at the center. Microbial communities deconjugate and transform bile acids, and the resulting pool signals to liver and intestine through FXR and TGR5. Intestinal FXR activation represses hepatic bile acid synthesis through fibroblast growth factor 15 (26, 27). Microbially derived short-chain fatty acids act on the same tissue. GLP-1 secretion is itself embedded in this circuitry, since bile acid activation of TGR5 in enteroendocrine L cells stimulates GLP-1 release in mice (28). The species level at which each step rests is the relevant qualification. Propionate reduced steatosis in male mice and in a hepatoma cell line at 0.5 to 1 mM. Its receptor dependence was inferred from a single antagonist, without genetic or *in vivo* confirmation (29). No human study has yet linked a treatment-induced change in microbial bile acid or short-chain fatty acid output to a hepatic histological outcome.

Whether incretin therapy itself acts through this relay is less settled than the mechanism suggests. Two randomized trials in type 2 diabetes bear on it and are set out in **Supplementary Note 10**. Neither licenses a strong reading, since both used glycemic endpoints and unchanged diversity indices are not evidence of unchanged composition. What the pattern shows is that bile acid metabolism responds more reliably to incretin therapy than community-level indices do (30, 31). The microbiome should therefore be read as a modifier of the relay, not as its principal effector. That case rests on the pharmacology, not on these two results. The GLP-1 receptor is expressed on host cells and not on bacteria, so the community sits structurally downstream of the drug target. The changes in intake and transit through which the drug alters it are necessary intermediates. A domain downstream of the intervention can still modify how much hepatic benefit a given amount of weight loss yields, which is the role assigned to it below.

Direct hepatic action can nonetheless be engineered, as the glucagon component of survodutide shows, so the newer agents combine an indirect, appetite-mediated route with a direct hepatic one by design.

The resulting picture is coherent in outline but unresolved in the detail most relevant to prediction. Hepatic benefit is delivered through overlapping systemic and gut–liver routes whose relative contributions vary between individuals. The unanswered question is not whether these pathways exist, but how their patient-to-patient variation maps onto observed differences in response. Two features of the circuitry are measurable before treatment and mechanistically tied to it: host genotype and the gut microbiome. They are the entry points for the framework developed below.

## 4 Host genetic determinants of disease and drug response

Host genetics offers what clinical phenotyping cannot. It supplies a set of determinants that are fixed at birth, measurable before the first dose, and mechanistically wired into the hepatic lipid pathways that metabolic drugs engage. Over the past 15 years, a small group of common variants has been established as the genetic backbone of MASLD. These variants shape susceptibility to steatosis, the risk of progression to fibrosis, and, increasingly, the response to intervention. They are the first of the two pretreatment domains on which a predictive framework can be built, and their evidence base must be read with a careful distinction in mind. Influencing disease is not the same as influencing drug response, and only the latter directly supports patient stratification.

### 4.1 Genetic architecture and aggregation into risk scores

The genetic architecture of MASLD rests on four loci, replicated to differing degrees (**Table 4A**). The founding observation came from a genome-wide scan of non-synonymous variants (n = 9,229) in the multiethnic Dallas Heart Study. It identified PNPLA3 rs738409 (encoding I148M) as strongly associated with hepatic fat and inflammation. The association survived adjustment for body mass index, diabetes status, alcohol use, and both global and local ancestry, and it tracked the ancestry-related differences in disease prevalence (32). An exome-wide study then identified TM6SF2 E167K, which raises hepatic triglyceride while lowering circulating lipids and reducing VLDL secretion (33). A genome-wide study of alcohol-related cirrhosis confirmed PNPLA3 and added MBOAT7 rs641738, implicating phospholipid remodeling in fibrogenesis (36).

**Table 4A:** Pre-treatment candidate markers: what can be measured before the first dose, and what each measurement currently supports: Host genetic variants.

Locus and variant	Protein consequence	Association with disease, as reported	Replication	Tier	Assay and specimen	Evidence that it modifies treatment response
*PNPLA3* rs738409	I148M	Hepatic fat content P = 5.9 × 10^-^¹^0^ and hepatic inflammation P = 3.7 × 10^-4^ in a multiethnic genome-wide scan of nonsynonymous variants (n = 9,229); association with hepatic fat retained after adjustment for BMI, diabetes status, alcohol use, and both global and local ancestry (32)	Replicated across cohorts and ancestries	1 for disease progression; **3 for response**	Allelic discrimination or panel genotyping; whole blood or saliva	Change in liver stiffness tracked weight change in risk-allele carriers but not in wild-type patients in one single-center retrospective cohort (dietary advice, liver stiffness endpoint, single ancestry, **no formal genotype-by-weight-change interaction test**) (10). Genotype-restricted trials of AZD2693 enrolled only 148M homozygotes and showed an unambiguous pharmacological effect (40). In cell models, the variant showed **less** response to resmetirom, a direction opposite to the stratification hypothesis (39)
*TM6SF2* rs58542926	E167K	Raises hepatic triglyceride content while lowering circulating lipids and reducing VLDL secretion; identified at exome-wide significance (33)	Replicated	1 for disease progression; **no response data**	As above	None reported
*HSD17B13* rs72613567:TA	Protein-truncating splice variant	Reduces risk of non-alcoholic cirrhosis by 26% in heterozygotes and 49% in homozygotes; in 2,391 human liver samples associated with reduced risk of **steatohepatitis but not of steatosis** (37)	Replicated	1 for disease progression; **no response data**	As above	None reported. The steatohepatitis-but-not-steatosis dissociation is load-bearing for endpoint-specific calibration (§6, §7) rather than for response prediction
*MBOAT7* rs641738	Altered MBOAT7 expression	Meta-analysis of 42 studies, 1,066,175 participants of whom 9,688 had liver biopsies: hepatic fat association small in magnitude (0.03 standard deviations); advanced fibrosis OR 1.22 (95% CI 1.03–1.45), emerging **only under a recessive model and only in adults of European descent**, with no effect detected in children (38)	**Contested**	**2**	As above	None reported
Genetic risk score (3–5 variants), ancestry-calibrated	Not applicable	Aggregate scores are reported to outperform single variants for progression and liver-related events (9)	Not established	**3 (proposed)**	Panel genotyping	**No study has reported the incremental discrimination (ΔAUC or net reclassification) of a genetic score added to a clinical model in MASLD, for progression or for response.** This is a gap in the field, not an omission of this review

Values in bold identify where the evidence departs from a claim about response to GLP-1-based therapy.

**Table 4B:** Pre-treatment candidate markers: what can be measured before the first dose, and what each measurement currently supports: Gut microbial features.

Feature	Taxonomic level as measured	Detection method	Direction reported	Tier	What limits its use
*Faecalibacterium* depletion	Genus	16S rRNA amplicon and shotgun studies surveyed collectively (43)	Depleted in MASLD	2	Reported with less consistency than its citation frequency suggests
*Akkermansia muciniphila* depletion	Species	As above	Depleted in MASLD	2	The interventional evidence uses a **pasteurized, non-viable** preparation, which improved insulin sensitivity by 28.6% in a 32-participant proof-of-concept trial with **no prespecified hepatic endpoint** and without altering community composition
*Escherichia*/*Escherichia–Shigella* enrichment	Genus; ***Escherichia–Shigella* is a merged operational label of 16S amplicon data**; the V3–V4 region cannot separate the two genera	16S rRNA amplicon	Enriched in MASLD; also enriched in lean MASLD alongside depletion of short-chain fatty acid producers	2	**Metformin produces the same direction of change** (48), so disease and background medication are confounded in any cohort not stratified by metformin exposure
*Prevotella*-to-*Bacteroides* ratio; *Prevotella* abundance	Genus	**16S rRNA-targeted qPCR** for the ratio studies; 16S amplicon and shotgun elsewhere	**Direction is not stable.** High ratio marked *better* fat loss on fiber (3.15 kg, 95% CI 1.55–4.76) (50) and *better* loss on whole grain (1.80 kg) (51), but *greater regain* during maintenance (52); high *Prevotella* marked *low* responders to resistant starch (12)	2	The originating group failed to reproduce its own stratification (53). *Prevotella copri* comprises clades differing by more than a 10th of their genomes with distinct polysaccharide utilization, present in 95.4% of non-Westernized versus 29.6% of Westernized individuals (54); strain-level heterogeneity has been advanced as the explanation for the field’s contradictory findings (55). **Genus-level reporting is itself part of the problem**
*Bacteroides stercoris* and related species tracking response to resistant starch	Species	Shotgun metagenomics	Tracked reduction in intrahepatic triglyceride (49)	2	Substrate-delivery intervention, not a drug
Baseline microbial plus clinical classifier of response	Genus and species	Shotgun metagenomics with multi-omic integration	AUC **0.74 (95% CI 0.66–0.81) on independent multicenter external validation**; 0.87 in the derivation trial, where feature selection and synthetic oversampling were performed (12)	**2 for response to a dietary prebiotic; 3 for response to a GLP-1 receptor agonist**	The transfer from prebiotic to drug is not a short step along a shared pathway: a substrate is used *by* the community, whereas a GLP-1 receptor agonist acts on **host** receptors and the community sits downstream of the drug target
Advanced-fibrosis classifier	Species	Whole-genome shotgun	AUC 0.936 (45)	2	Predicts stage of disease, not response to treatment
Alpha and beta diversity indices	Community level	16S rRNA amplicon	Unchanged by liraglutide in a 12-week randomized trial (30)	3	**Unchanged diversity indices are not evidence of unchanged composition.** Diversity has no detectable heritability (68)

Abundances are relative and therefore compositional: an apparent decrease in two taxa and increase in a third may be three projections of a single compositional shift rather than three independent findings. Tier assignments follow the admission criteria in **Table 2**. No marker in either part of this table is currently qualified to gate a prescribing decision; **Table 6** states, for each axis, the observation that would refute it.

Taxonomic level and detection method are stated for every row, because they are not interchangeable. Genus-level 16S assignments cannot resolve species or strains, and the clade structure within a single genus can reverse the direction of an association (see *Prevotella*, below).

Values in bold identify the limitation that currently disqualifies the marker from guiding a prescribing decision.

The causal mechanism of the PNPLA3 variant is established in animal and cellular models (34, 35) and is set out in Section 5.4 and **Supplementary Note 8**. The resulting steatosis does not require loss of catalytic activity. That is the basis for treating the allele as a substrate rather than only a marker, a point taken up below.

In contrast to these risk alleles, a protein-truncating splice variant in HSD17B13 (rs72613567:TA) is protective. It protects against progression from steatosis to steatohepatitis and reduces the risk of non-alcoholic cirrhosis by 26% in heterozygotes and 49% in homozygotes. In 2,391 human liver samples, it was associated with reduced risk of steatohepatitis but not of steatosis (37). That last detail matters beyond this section. Within one human dataset, it establishes that a hepatic exposure and a hepatic outcome can come apart under genetic control. That is the pattern on which the endpoint-specific calibration argued for below depends. Together, these loci converge on lipid handling within the hepatocyte, and each is stable and assayable long before therapy is considered.

The magnitude and consistency of these effects vary with ancestry and endpoint, which tempers any simplistic reading. The PNPLA3 risk allele is most frequent in Hispanic populations and least frequent in individuals of African ancestry, mirroring differences in disease burden (32). The secondary variants replicate less uniformly. A meta-analysis of 42 studies found the MBOAT7 association with hepatic fat small in magnitude (38). Its association with advanced fibrosis emerged only under a recessive model, and only in adults of European descent. **Table 4A** gives the magnitudes. This heterogeneity of effect, in magnitude and in the genetic model required to detect it, is precisely why single variants are insufficient for prediction. It motivates aggregation into genetic risk scores. These typically combine PNPLA3, TM6SF2, and HSD17B13 and predict steatohepatitis, fibrosis progression, and liver events more reliably than any single polymorphism (9). The trajectory of the field is therefore toward integrated scores calibrated for the population in question.

### 4.2 Genotype and drug response: a distinct evidence base

The evidence above establishes that genotype shapes disease. Stratifying treatment requires the distinct and less mature evidence that genotype shapes drug response. Here, the most direct data come from weight reduction rather than from a drug. A retrospective single-center cohort followed 140 Japanese patients with biopsy-proven fatty liver for 1 year after a dietary consultation. Change in liver stiffness correlated with weight change in PNPLA3 risk-allele carriers (r = 0.28 for CG, 0.38 for GG) but not in wild-type patients. A similar pattern held for the HSD17B13 rs6834314 minor allele (10). Three features limit what this establishes. The variant genotyped is a missense change, not the protein-truncating splice variant discussed above, so the two are not interchangeable. One HSD17B13 genotype was pooled because only 11 patients were homozygous, precluding any statement about allele dosage. The analysis compared correlations within strata without the formal genotype-by-weight-change interaction test, which the paper does not report. Read conservatively, carriers appear to extract a steeper reduction in liver stiffness per unit of weight lost, while losing weight less readily (2.9%, 1.3%, and 0.2% across CC, CG, and GG, respectively). Since GLP-1 hepatic benefit is itself largely delivered through weight loss, this genotype-dependent efficiency is a plausible determinant of incretin response. No genotype-stratified GLP-1 trial has demonstrated it. Experimental systems point the same way: in a genotype-defined liver microphysiology platform, cells carrying PNPLA3 I148M responded differently from wild-type cells to several metabolic drugs (39).

### 4.3 Risk variants as therapeutic targets

That the same genes are now becoming drug targets reinforces their causal centrality while exposing the current translational ceiling. Genotype-directed silencing has reached early-phase clinical testing for both loci. AZD2693, a liver-targeted antisense oligonucleotide against PNPLA3, was tested in two randomized phase 1 trials restricted to 148M homozygotes. It achieved 89% knockdown of hepatic PNPLA3 mRNA, and placebo-corrected reductions in liver fat of 7.6% and 12.2% at the 25- and 50-mg doses over 12 weeks (40). On the protective locus, the RNA-interference agent ARO-HSD reduced hepatic HSD17B13 mRNA by a mean of 78.6%, with accompanying reductions in alanine aminotransferase (41). A subsequent phase 1 study of an RNA-interference therapeutic against HSD17B13 in MASH confirmed target engagement and tolerability (42). These trials are notable for a design feature directly relevant to this review. Enrollment was itself genotype-defined, which makes them the first examples of genotype-stratified drug development in this disease. Yet, despite the robustness of the underlying genetics, no MASLD-associated variant has entered routine clinical use for guiding the choice of therapy. The gap between prognostic association and actionable prediction remains the principal obstacle.

### 4.4 Established, missing, and unresolved

The genetic pillar can thus be summarized. What is established is a compact, ancestry-sensitive set of variants that govern hepatic lipid handling and disease progression, best deployed as genetic risk scores rather than single markers. What is missing, and the study designs that would supply it, are set out in **Table 5**. The gut microbiome, whose signaling intersects the very lipid pathways these variants control, is the subject of the section that follows.

**Table 5:** Evidence gaps, the tier they affect, and the study design that would close them.

#	Evidence gap	Tier affected	Study design that would close it	New recruitment required
1	No prospective evidence that risk variants predict response to GLP-1–based therapy specifically, as opposed to weight-loss response in general	Tier 3 → tier 1	Genotype-stratified reanalysis of completed incretin trials, with a formal genotype-by-weight-change interaction test	No: existing trial biospecimens
2	Genetic risk scores are not calibrated across ancestries, risking inequitable prediction	Tier 2	Recalibration in ancestry-diverse cohorts with reporting of absolute risk by percentile	No: existing cohorts
3	No quantitative incremental discrimination reported for any genetic score in this disease	Tier 2	Nested models reporting change in the area under the curve and net reclassification over a clinical baseline	No: existing cohorts
4	Net effect of an allele that raises hepatic return per kilogram lost while reducing kilograms lost has never been reported	Tier 3	Report weight-change distribution and total hepatic change by genotype, with formal mediation analysis	No: existing trial data
5	No demonstration that baseline microbiota predicts response to GLP-1–based therapy, as distinct from response to a delivered substrate	Tier 3 → tier 2	Baseline microbial profiling of stored samples from completed incretin trials, with response as the prespecified endpoint	No: stored samples, where collected
6	Microbiome assays lack standardization, reference ranges, and clinical decision thresholds	Tier 3	Multisite analytical validation across extraction, sequencing, and database versions, with prespecified thresholds	Prospective
7	Causality in humans rests on trials with intermediate endpoints; genetic instruments for microbial traits are too weak to adjudicate	Tier 3	Transplantation trials powered for hepatic endpoints; larger microbiome genome-wide association studies to strengthen instruments	Prospective
8	Complementarity of host and microbial information has never been quantified	Tier 2	Nested host, microbial, and combined models in a cohort whose outcome is response to therapy	No: existing cohorts
9	No trial stratifies enrollment by genotype or baseline microbiota with cardiovascular events as a primary endpoint	Tier 3	Prospective stratified trial, or individual participant data meta-analysis of cardiovascular outcome trials with genotype available	Prospective
10	No fibrosis-risk instrument validated for cardiovascular rather than hepatic prediction; no direct genetic test of whether fibrosis contributes independently to cardiovascular events	Tier 3	Instrument development in cardiovascular outcome cohorts; instrumental-variable analysis with fibrosis rather than hepatic fat as the exposure	No: existing consortium data
11	Whether the lean phenotype is a distinct stratification unit or a low-adiposity subset of the same framework is untested	Tier 3	Prospective subtype cohorts following clinical outcomes, with formal interaction testing by adiposity stratum	Prospective
12	No pretreatment variable is known to predict hepatic and cardiovascular benefit concordantly	Tier 3	Joint modeling of both endpoints in a single stratified cohort	Prospective

Gaps are grouped by the domain in which they arise. The final column states whether the question can be answered from existing biospecimen collections or requires prospective recruitment, which is the practical determinant of how soon each tier can advance.

## 5 Gut microbiome signatures and their predictive value

The gut microbiome is the second pretreatment domain on which stratification can be built, and it complements host genotype. Where genetic variants are fixed, microbial communities are modifiable, which makes them both a candidate biomarker and a potential therapeutic lever. Its promise for prediction, however, rests on evidence of uneven maturity. A recurring but only partly reproducible disease-associated signature exists and appears to carry fibrosis-stage information, although cross-cohort inconsistency and methodological noise complicate its interpretation. The decisive question is whether baseline composition predicts who responds to therapy, and it rests so far on proof of concept rather than drug-specific human trials. Each stratum must be read on its own terms.

### 5.1 A recurring signature that tracks fibrosis stage

A recurring compositional signature distinguishes MASLD from health, although with less consistency than the frequency of its reporting suggests. Across the clinical sequencing and metabolomic studies surveyed, the disease has been marked by depletion of *Faecalibacterium* and *Akkermansia muciniphila* and by enrichment of *Escherichia*. Shifts in bile acid, short-chain fatty acid, and tryptophan metabolites accompany these changes (43) (**Tables 4**). The largest attempt to test how much of this survives harmonization pooled public metagenomic data from 1,206 subjects with and without metabolic disease. It built classifiers that were portable across cohorts and specific to MASLD rather than to obesity, type 2 diabetes, hypertension, or atherosclerosis, with accuracies of 0.845 to 0.917. Its authors concluded that defined microbial consortia, rather than individual species, are the realistic unit for both signature and therapeutic development (44). That conclusion is a caution about the taxon lists reproduced above, not a confirmation of them.

More consequential than the case–control contrast is whether the microbiome tracks disease severity. A prospective whole-genome shotgun study derived a classifier that detected advanced fibrosis with an area under the curve of 0.936 (45). That figure should be read with its cohort: 86 patients, only 14 with advanced fibrosis, and 40 features of which 37 were bacterial species. Fitting 37 features to 14 events invites optimistic bias, the classifier was not built on stool composition alone, and the external validation that would counter this has not been performed. The authors described the result as preliminary evidence, and it is best cited that way. Two features of this severity association bear on whether the community is coupled to disease activity or merely co-varies with it. The first is that the taxa tracking progression are the ones that generate the metabolites relayed to the liver. Depletion of *Faecalibacterium* removes a major producer of butyrate. The accompanying bile acid and short-chain fatty acid shifts (43) act on the receptors through which the gut–liver axis operates. These are FXR and TGR5 for the bile acid pool (26, 28), and G-protein-coupled receptors for the short-chain fatty acids (29). The signature is thus not an arbitrary taxonomic list that happens to correlate with stage. Its constituents sit on the pathway by which intestinal events reach the hepatocyte. The second feature is that severity association does not establish direction. A community shift could drive fibrogenesis, follow from the altered bile acid pool of advancing disease, or track a third factor such as diet. The evidence in Section 5.4 bears on that question and does not resolve it in favor of the community being causal. What the association does establish is weaker but sufficient here: the community carries stage information, and it sits on the relay through which incretin benefit is delivered. That is what raised the possibility that it might also carry information about treatment response.

### 5.2 The limits of reproducibility

The strength of this signature should not be overstated, because its reproducibility is bounded by real biological and methodological heterogeneity. Alpha diversity is the clearest case. It is frequently reported as reduced in MASLD, but it is not a stable disease feature. The direction is inconsistent across studies, and the value depends heavily on sequencing depth, rarefaction strategy, and which index is chosen. The elastography-staged cohort discussed below found no difference in alpha diversity between fibrosis groups at all, with separation confined to beta diversity (46). It should not be counted among the features that define the disease.

The harmonized reanalysis cited above exposes how much of the apparent between-study disagreement stems from non-comparable cohorts and analytic choices rather than from biology. Its finding that consortia outperform individual species implies that taxon-by-taxon signatures will remain unstable (44). Reported discrimination in the 0.9 range, in this literature, derives from derivation cohorts of modest size without external validation. It should be read as apparent, not expected, performance. Any predictive use of the microbiome must therefore be built on features that survive harmonization rather than on single-cohort associations. It must also contend with stool composition being an indirect readout of the tissue-level and metabolite-level processes that actually matter.

Drug exposure is a first-order confounder of the MASLD microbiome, and metformin is the clearest instance (**Supplementary Note 1**). It produces reproducible compositional change in treatment-naive type 2 diabetes. Transfer of metformin-altered microbiota to germ-free mice improves glucose tolerance, so the effect is consequential rather than incidental (47). In non-diabetic obese women, it specifically increased *Escherichia*/*Shigella* abundance (48), the same direction of change attributed above to disease. Metformin is taken by a large fraction of patients with coexisting diabetes, which is itself one of the axes this review proposes to use. The features most often cited as a MASLD signature therefore cannot presently be separated from the pharmacological signature of the drug most of these patients are taking.

### 5.3 From association to prediction of response

The question is not whether the microbiome reflects disease but whether it predicts response, and here the evidence makes a genuine but circumscribed advance. The relevant experimental line is the resistant-starch trials, set out at full resolution in **Supplementary Note 9**. A randomized placebo-controlled trial in MASLD identified baseline microbiota as the dominant determinant of response heterogeneity, by multi-omic analysis and by transfer of patient microbiota into antibiotic-depleted mice (12). High *Prevotella* abundance marked low responders, and coculture and transfer supported a causal role for the genus rather than stratification alone. A model integrating baseline microbial and clinical features reached an area under the curve of 0.74 (95% CI 0.66 to 0.81) in independent multicenter validation (12). That external estimate is the one cited here. This is the strongest demonstration that pretreatment microbial features can forecast response to a metabolic intervention in MASLD.

Its limits must be stated with equal clarity, and they are structural rather than a matter of the drug not yet having been tested. Resistant starch is a substrate delivered to the community, so baseline fermentative capacity lies causally upstream of the response. A community that cannot use the substrate is a mechanism of non-response. A GLP-1 receptor agonist, by contrast, acts on host receptors. Microbial change follows from reduced intake, slowed transit, and weight loss rather than preceding the effect. There is no equivalent notion of a community being unable to use the drug. Describing the two as sharing a gut–liver signaling route states something true while concealing the difference that matters. The inference from one to the other is not a short step along a common pathway. It is a transfer between two causal architectures, in which the microbiome occupies opposite positions relative to the intervention. The extrapolation may still hold, since a community shaping hepatic metabolite exposure could modify how efficiently weight loss is converted into hepatic benefit. Yet, that is a claim about effect modification, not about baseline prediction, and it has not been tested.

A second limit concerns the marker itself, and the microbiome literature has already documented it in the opposite direction. In fiber and whole-grain interventions, a high *Prevotella* to *Bacteroides* ratio has repeatedly marked the better metabolic responders rather than the worse (50, 51). The same ratio marked greater regain during maintenance (52), and **Table 4B** gives the magnitudes. The discrepancy cannot be resolved by declaring the genus beneficial or harmful. The group that first proposed the stratification later failed to reproduce it (53), and reporting at the genus level is itself part of the problem. *Prevotella copri* comprises clades that differ by more than a 10th of their genomes and utilize distinct plant polysaccharides (54). Strain-level heterogeneity has been advanced as the explanation for the field’s contradictory findings (55). The operational consequence for the framework proposed below is specific. A marker defined at genus level, in one dietary context and one population, cannot be carried unchanged into a different drug, endpoint, and background diet.

A third limit concerns the specimen rather than the taxon. Stool samples the distal colonic lumen. Intestinal FXR activation and bile acid reabsorption occur in the terminal ileum (26, 27), and the mucosa-adherent community is not the one a fecal sample captures. A fecal profile is thus a surrogate for the compartment of interest, and sampling that compartment directly requires endoscopy. This does not disqualify the specimen. A predictive marker needs only to share an upstream cause with the response mechanism, and the resistant-starch model was both built and externally validated on stool (12). What it argues for is a nested validation design (**Supplementary Note 3**). Until that comparison exists, a fecal predictor is an empirical marker under test rather than a measurement of the mucosal interaction.

### 5.4 Mechanisms of progression, the genotype–microbiome interaction, and what remains missing

On the host side, these variants are not markers of progression but participants in it. PNPLA3 I148M accumulates on the lipid droplet by evading ubiquitylation, and knock-in animals develop steatosis (34, 35). The retained lipid is droplet-centered, leaving very-low-density lipoprotein production and clearance indistinguishable between genotypes (80). The variant therefore governs how the hepatocyte disposes of incoming lipid rather than how much arrives, and **Supplementary Note 8** sets out the droplet biology. HSD17B13 rs72613567 marks the complementary step: its protein-truncating allele protects against steatohepatitis and cirrhosis without protecting against steatosis (37), placing its effect on progression rather than on fat accumulation.

On the microbial side, the taxa that track stage are the ones generating the metabolites relayed to the liver. Depletion of butyrate producers and the accompanying bile acid shifts (43) act through FXR and TGR5 (26, 28) and through enterohepatic FGF15 signaling (27). Short-chain fatty acid receptors driving hepatic lipolysis are a third route (29). That the community contributes rather than merely accompanies is established in animals. Germ-free mice receiving microbiota from divergent donors developed comparable obesity on an identical diet yet diverged in hepatic phenotype (66).

The interplay follows from where each mechanism sits. The microbial arm governs the metabolite and lipid exposure arriving at the hepatocyte. The genetic arm governs what the hepatocyte then does with it. A relation of that shape is multiplicative rather than additive, so it should appear as effect modification and not as two independent contributions. This is the mechanistic reason for entering both domains as modifiers in Section 6.4. It is also why the quantity still missing is an interaction term rather than a second main effect (**Supplementary Note 11**).

Microbial prediction cannot be considered in isolation from host genetics, but the evidence that the two are complementary is weaker than often assumed. In a 16S study of 156 patients staged by magnetic resonance elastography, multivariable analysis associated bacterial genera, PNPLA3 genotype, older age, and diabetes independently with advanced fibrosis. A random-forest classifier reached an area under the curve of 0.93. That classifier, however, was built on three genera alone, and the study reported neither a genotype-only nor a combined host–microbial model (46). The incremental discrimination gained by adding genotype to microbial features has therefore not been estimated, there or anywhere else. What the study does establish is that host and microbial variables are independently associated with the same hepatic outcome, which makes complementarity a reasonable expectation rather than a measured quantity. The distinction matters, because the case for combining the two domains rests on that expectation and not on a demonstration.

The microbial pillar can be summarized by separating what is secure from what is aspirational. What is established is a recurring disease signature that carries fibrosis-stage information and, in one controlled setting, predicts response to a dietary intervention. What is not established, and is often assumed, is that microbial and genetic information have been shown to be complementary in the quantitative sense a model requires.

In humans, the interventional record is more sobering than the associative one, and it is assembled in full in **Supplementary Note 7**. Its shape is easily stated. The largest trial randomized 104 patients to a synbiotic, altered the fecal microbiome, and moved neither liver fat nor either of two validated fibrosis scores. Weight change was the only variable independently associated with a decrease in liver fat (56). Smaller probiotic studies did not survive adjustment for weight change (57, 58). Three randomized trials of fecal microbiota transplantation reported no improvement in hepatic fat or insulin sensitivity, and the single positive trial was open-label against oral probiotics (59–62). Meta-analysis across 21 trials found reductions in alanine aminotransferase and liver stiffness with heterogeneity above 90% (63). Only two microbiome-targeted trials have since made liver histology their primary endpoint, enrolling 14 and 39 patients (64, 65). The field therefore has no adequately powered histological test of microbial manipulation, and the one trial powered for quantitative hepatic endpoints showed a negative result.

Causal inference about the microbiome rests on three tiers of evidence that do not converge. Colonization experiments remain the strongest: germ-free animals receiving microbiota from divergent donors developed comparable obesity on a high-fat diet yet diverged in hyperglycemia and hepatic phenotype (66). Transplantation trials in patients rank above every observational microbiome study on causal grounds, and as set out above, they have shown negative results for hepatic endpoints. Mendelian randomization, supportive elsewhere in this review, returned nothing robust here: across 1,198 tests, no association survived correction for multiple testing (67). That is not refutation, because the instruments are intrinsically weak. In the largest host genetic study of the microbiome, only the lactase locus reached study-wide significance (68), which places a low ceiling on the variance any microbial instrument can explain. The asymmetry is interpretable: genetic instruments are strong enough to interrogate host lipid biology and too weak to interrogate the community.

The distinction that governs this section is between a predictive marker and a therapeutic lever, which make different demands. A marker needs only to share an upstream cause with the response mechanism to carry predictive information. A lever must lie on the causal path and be manipulable in the intended direction, so the former does not imply the latter. The microbial evidence in this section illustrates the gap precisely. Baseline composition forecast response to a substrate delivered to the community. Deliberate manipulation of that composition failed to move hepatic endpoints in the trial designed to detect it.

What is missing follows from that gap and is listed with the corresponding study designs in **Table 5**. Before integration is addressed, the lean MASLD phenotype offers a natural test case in which genotype and microbiome interact with unusual clarity.

### 5.5 Adiposity independence of the genetic axis: evidence from lean MASLD

Lean MASLD subtracts the confounder that obscures both of these domains. In this phenotype, steatosis arises below a body mass index of 25 in Western populations and 23 in Asian ones. That leaves the residual contributions of genotype and microbial composition easier to discern. The phenotype is neither rare nor benign, and its outcomes are at least as serious as those of its obese counterpart (69). That makes it an informative limiting case, not an exception.

The expectation that genotype matters more once obesity is removed has been tested directly, and it was not confirmed. The study staged 312 patients across body mass index categories, with validation in an independent Latin American cohort. The prevalence of significant fibrosis did not differ between lean and non-lean disease (27.3% versus 31.1%). A high genetic risk score was associated with more significant fibrosis (36.5% versus 25.2%), and to a similar degree in both groups (70). The correct reading is more useful than the expected one. Genotype predicts fibrosis largely independently of adiposity, which broadens rather than narrows the case for genotype-based stratification. The lean phenotype is therefore useful as a setting in which the two domains can be observed without the confounding of obesity. It is not a subgroup in which they act more strongly.

The microbial contribution rests on thinner evidence. The signature reported in lean MASLD repeats the pattern described in Section 5.1. A combination of taxa has been reported to discriminate lean disease in Indian populations, although the supporting synthesis reports no discrimination metric (71). These features have been read as combining with host polymorphisms to determine whether steatosis develops at normal weight (72). No study has tested that interaction formally here, so the claim is a hypothesis about the lean phenotype rather than a finding within it.

Read as a test case, lean MASLD constrains the central argument of this review as much as it supports it. It shows that when adiposity is held constant, genotype and microbiome remain operative determinants of disease. It also defines a concrete gap. The lean phenotype has been excluded or underrepresented in the pivotal drug trials, so no evidence indicates whether GLP-1–based therapy performs in lean as in obese disease. Prospective subtype cohorts following clinical outcomes are beginning to supply the comparative data (73). That transfer remains open, and it is one of the constraints the framework in the next section must accommodate.

## 6 Toward an integrated precision-medicine framework

The preceding sections leave several partial signals that have never been assembled. Response to incretin therapy is heterogeneous and has been explained only retrospectively by weight loss. Host genotype is a fixed determinant of hepatic lipid handling, and microbial composition is a modifiable one positioned along the indirect route to the liver. In the lean phenotype, both remain operative where metabolic phenotyping fails. The constituent evidence differs sharply in maturity, and that difference determines what each finding may be used for. The framework is therefore set out in three tiers, with admission criteria given before any finding is assigned (**Table 2**). The arrangement has a consequence a topic-based one would conceal. No item in the top tier concerns prediction of response from genotype or from baseline microbiota, because no such item currently meets the criteria (**Figure 3**).

**Figure 3:**
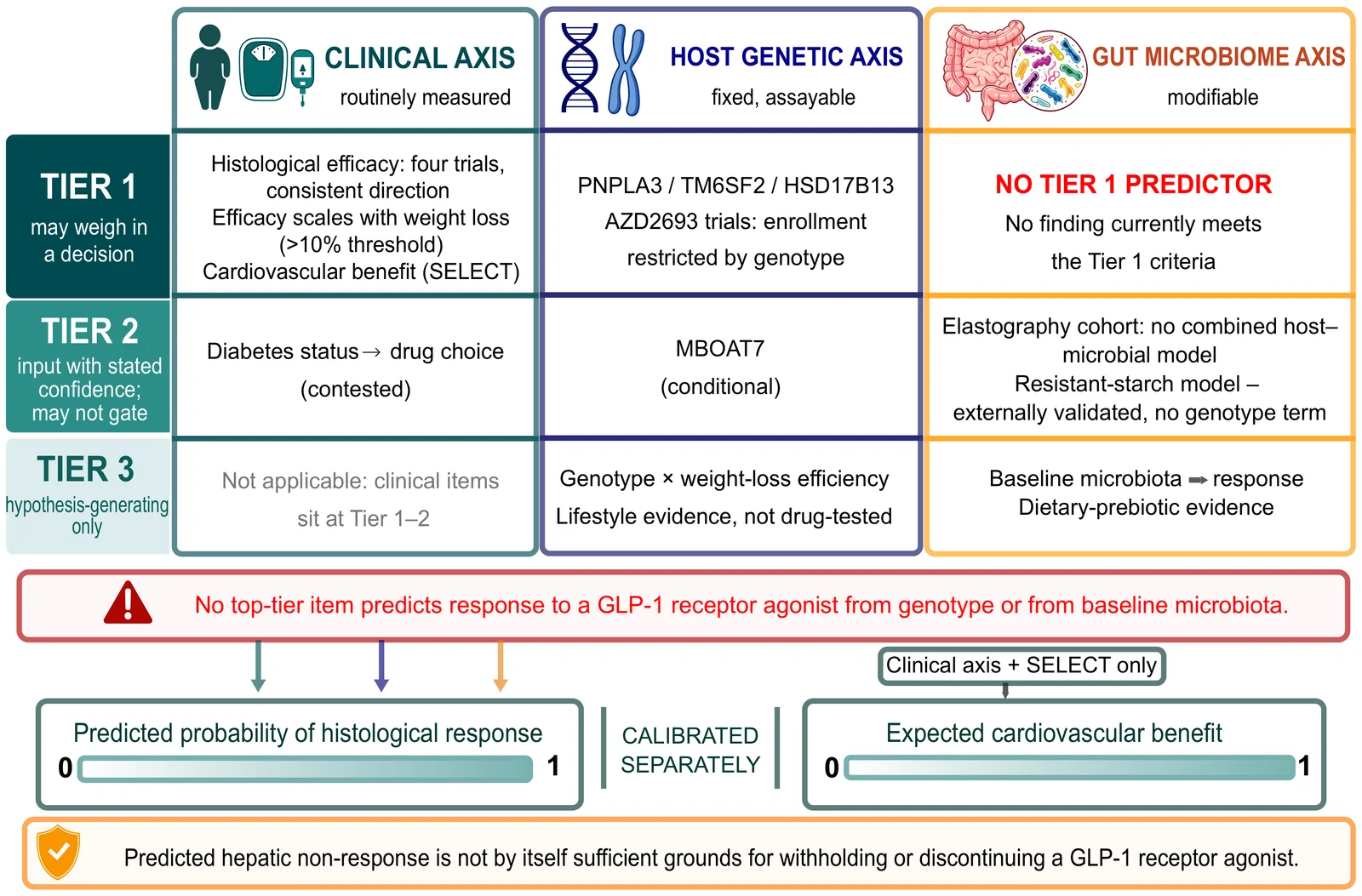
An evidence-tiered, endpoint-specific framework, and the cell it cannot yet fill. Candidate pretreatment inputs are arranged by the axis on which they are measured and by the maturity of the evidence supporting them. Admission criteria for the three tiers are given in **Table 2**. Tier 1 findings may carry weight in a stratification decision. Tier 2 findings enter as inputs carrying an explicit confidence estimate and may not gate a decision. Tier 3 findings generate hypotheses only. The host genetic and gut microbial axes are shown unranked. The cell marked as empty is the point of the figure. No finding currently meets the tier 1 criteria for predicting response to a GLP-1 receptor agonist from baseline microbiota. No item in the top tier predicts that response from genotype either. The two outputs are calibrated separately, because expected cardiovascular benefit is not calibrated by the hepatic axis (**Figure 4**). Both are continuous probabilities rather than a division into responders and non-responders. One rule is already actionable: predicted hepatic non-response is not by itself sufficient grounds for withholding or discontinuing a GLP-1 receptor agonist.

### 6.1 Tier 1: replicated in humans for a hepatic endpoint

Tier 1 admits findings reproduced in at least two independent human cohorts, or tested within a single randomized trial. The direction of effect must be consistent on a hepatic histological or validated hepatic outcome. Findings in this tier may carry weight in a stratification decision. Five items qualify.

The histological efficacy of incretin-based therapy is consistent in direction across four trials spanning a decade and three agents (6, 7, 15, 16). The dependence of that efficacy on the magnitude of weight loss is reproduced at the study level. It appears both in meta-regression across interventions and in a subgroup meta-analysis in which fibrosis benefit was confined to trials achieving at least 10% weight loss (21, 22). The association of PNPLA3, TM6SF2, and HSD17B13 variants with disease progression is replicated across cohorts and ancestries (32, 33, 36, 37). The fourth commonly cited locus, MBOAT7, does not clear this threshold and is placed in tier 2 below. Cardiovascular benefit from semaglutide is established in a large outcome trial and is consistent across strata of a biochemical fibrosis-risk index within it (74, 75).

The fifth item is the least remarked and the most consequential for what follows. The genotype-restricted AZD2693 trials set out above enrolled only 148M homozygotes and produced an unambiguous pharmacological effect in them (40). A trial that stratifies enrollment by genotype is therefore not a new design in MASLD but an existing one awaiting application to a different drug class.

What tier 1 does not contain is the proposition on which stratification by host biology depends. No finding linking genotype or baseline microbiota to response to a GLP-1 receptor agonist meets these criteria. The framework below is built in the knowledge of that absence rather than around it.

### 6.2 Tier 2: contested, or single-study without external validation

Tier 2 admits human evidence on a relevant endpoint that is inconsistent across studies, or derived from one cohort without independent replication. It also admits evidence that depends on the significance of separate subgroups rather than on a formal test of interaction. Findings here may enter the framework as inputs carrying an explicit confidence estimate. They may not gate a decision.

The first item is the variable clinicians can already measure. A meta-analysis of three placebo-controlled histological trials in non-cirrhotic MASH reported a reciprocal pattern. Among patients with type 2 diabetes, semaglutide did not improve fibrosis stage (RR 1.21, 95% CI 0.94–1.56) whereas resmetirom did (RR 1.99, 95% CI 1.35–2.93). Among patients without diabetes, the direction reversed, with semaglutide improving fibrosis (RR 1.72, 95% CI 1.21–2.44) and resmetirom not (RR 1.37, 95% CI 0.83–2.25) (76). Taken at face value, a single routinely available variable would govern drug choice. However, the comparison is across trials rather than within them and rests on separate subgroup significance instead of a formal interaction test. The source populations also differ in baseline fibrosis, trial duration, and background glucose-lowering treatment, any of which can generate the same pattern. Metformin exposure is a further systematic difference, acting on the microbial axis as well (47). Two larger syntheses disagree with this reading. A meta-analysis of 13 randomized trials found GLP-1 receptor agonists superior to placebo for both endpoints regardless of diabetes status, with no detectable between-study heterogeneity (77). Its histological comparison rested on three of them. A network meta-analysis of 69 trials cautioned that subgroup evidence remains shallow (78). The best-measured axis is the least settled, and no framework can resolve this by construction.

The second and third items concern whether host and microbial information are complementary. Each is placed here by a single feature: the elastography cohort reported no combined host–microbial model (46), and the resistant-starch trial included no genotype term (12). Establishing complementarity requires what neither reports: nested models yielding incremental discrimination and reclassification, in a cohort whose outcome is response to therapy rather than stage of disease.

A fourth item is one of the four commonly cited risk loci itself. On meta-analysis of 42 studies, the MBOAT7 variant rs641738 was associated with advanced fibrosis only under a recessive model of inheritance and only in adults of European descent. No effect was detected in children, and its association with hepatic fat was small in magnitude (38). It is therefore an input whose contribution is conditional on ancestry and on the genetic model assumed. It cannot carry the weight in a stratification decision that the three tier 1 loci can.

Concomitant medication constrains the microbial axis more severely. The constraint is structural, not adjustable. Most patients with MASH take several drugs that alter the gut community, metformin being the clearest case (47, 48). A pretreatment profile in such a patient mixes disease, drug, and diet. Covariate adjustment cannot separate them: a binary medication term treats a mediated pathway as a confounder, while adjusting for the features it alters conditions on an intermediate. Three designs avoid the problem rather than model it, of which only prespecified stratification by regimen at enrollment identifies the signature cleanly (**Supplementary Note 1**). This is the most concrete reason the microbial axis remains in tier 2 and tier 3, rather than a reflection of insufficient sequencing effort.

### 6.3 Tier 3: inferred by extrapolation

Tier 3 admits evidence transferred across drug class, endpoint, or population, or from an experimental system to a patient. Findings here specify trial designs and generate hypotheses. They carry no weight in a stratification decision.

The genotype dependence of hepatic benefit sits here, on the single-center retrospective cohort in Section 4.2, which is the whole human evidence for it (10). The features bounding the inference are set out in Section 4.2, and its exposure and endpoint are recorded in **Table 1**. The absence of a formal interaction test is the same limitation for which the diabetes signal sits in tier 2, applied here for the same reason. The net effect of the allele has never been reported, and the threshold behavior set out earlier makes its sign consequential rather than academic. If fibrosis benefit is largely confined to patients achieving at least 10% weight loss, an allele making that threshold harder to reach could offset a steeper hepatic return entirely. Reporting weight-change distribution and total hepatic change by genotype, with formal interaction and mediation analysis (**Supplementary Note 2**), would settle it from data that already exist.

The genotype-defined microphysiology system complicates the simple reading rather than supporting it, since the variant cells reproduced the clinical phenotype yet showed *less* response to resmetirom (39). **Table 1** records the exposure, and genotype is not separable from donor background in this design. Read conservatively, genotype can modify drug response in hepatocytes whereas the direction of that modification stays agent-specific. A variant might raise the hepatic return on weight loss while lowering the return on a directly acting hepatic drug. That possibility is a reason for the framework to address drug selection rather than response alone.

Transfer of microbial prediction to incretin therapy also sits here, for the structural reason set out above. The community lies upstream of a delivered substrate but downstream of a drug acting on host receptors. The two are therefore not a short step apart along a shared pathway. Consistent with this, the human trials examining incretin treatment directly found bile acid metabolism to respond more reliably than community composition did (30, 31).

### 6.4 Assembling the three axes

Three axes follow, and the tier assignment fixes the role each may play. A clinical axis comprising diabetes status, baseline fibrosis stage, and degree of adiposity supplies the baseline model. These are the only inputs standing in a tier 1 or tier 2 relationship to a therapeutic outcome. A host genetic axis and a microbial axis enter as candidate modifiers of the efficiency with which weight loss is converted into hepatic benefit. Treating both as modifiers rather than as independent predictors follows from the mechanism, not from convenience. Hepatic benefit is relayed through weight loss and gut–liver signaling, and both domains act on the relay rather than on the liver.

The specification of each axis should not outrun its evidence. The genetic axis is measurable today as PNPLA3 and HSD17B13 genotypes. Current cohorts sum PNPLA3 and TM6SF2 risk alleles minus the protective HSD17B13 genotype (70). That subtraction is not merely arithmetic. In one human dataset, the protective variant mitigated the injury associated with the PNPLA3 risk allele (37). These are oligogenic rather than genome-wide scores, and their principal portability problem is calibration, not discrimination (**Supplementary Note 6**). No incremental discrimination has been reported for any such score in this disease (9), a gap in the literature, not an omission here. The microbial axis is measurable today as taxonomic composition, and as targeted quantification of bile acids, short-chain fatty acids, and tryptophan metabolites. Read functionally, it is a specification, not a demonstrated predictor, since every microbial finding assembled above is taxonomic.

Reading the axis functionally can be made concrete. Candidate assays order themselves by how closely each approaches microbial activity rather than identity, from gene-level targets through metatranscriptomics to targeted metabolomics. The last sits closest to function and is the most tractable to implement (**Supplementary Note 4**). The differential responsiveness of bile acid metabolism noted above is itself an argument for assaying the metabolic output of the community rather than its membership.

Whether the microbial axis can serve as a target as well as a marker rests on narrower evidence than the framework’s rhetoric might suggest. In the resistant-starch trial, a *Bifidobacterium pseudocatenulatum* strain isolated from a high responder degraded resistant starch *in vitro*. Given to mice colonized with microbiota from low-responding patients, it reduced fecal residual starch and improved hepatic histology (12). The authors state this as a possibility, not a finding, and the strain has never been given to a patient. Set against the negative interventional record assembled earlier, the honest position is narrow. The microbial axis is the only one of the three for which a target has been identified at all. That target remains a hypothesis with preclinical support rather than a clinical option.

Where the axes disagree, the framework does not rank genotype above microbial features. The criterion that should govern such a ranking is how directly each domain has been tied to a therapeutic outcome in humans. By that criterion, the microbial evidence is the stronger. It comes from a randomized placebo-controlled trial with response as its endpoint. The genetic evidence comes from an observational cohort given a non-pharmacological intervention, with liver stiffness as its endpoint. Ordering them requires a criterion the present evidence cannot supply, so they are left unranked. The clinical axis is placed first on narrower grounds: contested as its interaction with drug class is, its inputs are the only ones measured in the trials that define efficacy.

### 6.5 Output form, refutation conditions, and validation requirements

**Table 6** sets out each axis with its inputs, assay, tier, role in the model, and the observation that would refute it. The last column is operative: an axis with no refutation condition cannot be evaluated.

**Table 6:** Operationalization of the three axes.

Axis	Inputs measurable today	Assay and specimen	Tier	Role in the model	Observation that would refute the axis
Clinical	Type 2 diabetes status; baseline fibrosis stage; BMI and waist circumference	Routine clinical data; biopsy or validated non-invasive fibrosis staging	1 (association with efficacy)/2 (interaction with drug class)	Baseline model	Formal interaction testing on individual participant data shows no modification of drug-specific fibrosis response by diabetes status
Host genetic	PNPLA3 rs738409; HSD17B13 genotype. *Proposed:* 3–5 variant genetic risk score, ancestry-calibrated	Allelic discrimination or panel genotyping; whole blood or saliva	3 for response; 1 for disease progression	Candidate modifier of the efficiency with which weight loss is converted into hepatic benefit	Genotype-stratified reanalysis of completed incretin trials shows <5 percentage-point difference in fibrosis improvement between 148M carriers and wild type after adjustment for weight lost
Gut microbial	Genus- and species-level relative abundance; targeted bile acid, short-chain fatty acid, and tryptophan metabolite quantification. *Proposed:* functional pathway and strain-level readouts	16S rRNA or shotgun metagenomics with a frozen database version; targeted mass spectrometry; stool and serum	3 for incretin response; 2 for response to a dietary prebiotic and for association with stage	Candidate modifier of the gut–liver limb of the relay; the only axis that is also a candidate target	Baseline microbial features add <0.05 to the area under the curve of a clinical model predicting histological response in stored trial samples

No axis in this table is currently qualified to gate a prescribing decision. The table specifies what would have to be measured, and what would have to be observed, for that to change.

Three properties of the output follow from the tier structure. Prediction should be probabilistic, not binary, since assigning non-responder status on evidence of this maturity would withhold effective therapy from patients whose expected benefit is merely uncertain. Output should address drug selection rather than response alone. The clinically useful question is which agent suits a given profile, and genotype may modify response in opposite directions for different drug classes. Any drug-selection output is further constrained by an asymmetry in the outcome evidence. Substituting a thyroid hormone receptor-β agonist for an incretin has no cardiovascular outcome data behind it. That constraint is stronger than a caveat. No alternative agent in MASH carries cardiovascular endpoint evidence, and the negative rule set out below forbids withdrawing a GLP-1 receptor agonist on predicted hepatic non-response. The drug-selection function therefore cannot be enabled at present. It is specified so that it can be switched on when comparative outcome data exist, not because it is available now.

The framework is stated so that it can fail. Three predictions carry it, each with a direction and a magnitude, and each answerable either without new recruitment or with a single stratified trial.

First, completed incretin trials can be reanalyzed with stratification by genotype. Suppose fibrosis improvement differs by less than five percentage points between PNPLA3 148M carriers and wild-type patients, after adjustment for weight lost. The genetic axis then fails as an efficiency modifier and should be removed. Second, baseline microbial features may add less than 0.05 to the area under the curve of a clinical model predicting histological response, in stored samples from those trials. The microbial axis would then fail as a predictor of response, whatever its association with stage of disease. Third, formal interaction testing on individual participant data may show no modification of drug-specific fibrosis response by diabetes status. The drug-selection function would then lose its only currently measurable input, and the framework would reduce to a probability of response to a single class.

The first of these predictions requires a specific analytic form, because the genetic axis carries an effect in each direction. Counterfactual mediation supplies it, with genotype as exposure, weight lost as mediator, and hepatic improvement as outcome. It separates a natural indirect effect transmitted through weight loss from a natural direct effect capturing the residual return per kilogram. The quantity that decides whether the axis is worth measuring is their sum, which may be null while both components are large. The decomposition and its identifying assumptions are set out in **Supplementary Note 2**. It requires no new recruitment: trials with stored DNA, serial weights, and paired biopsies contain what it needs.

The evidence that would advance each tier, ordered by feasibility, is set out with its study designs in **Table 5**. The agenda following from it is in Section 8. Until those requirements are met, the framework specifies what precision therapy in MASH would require rather than what it delivers. That leaves an immediate question: what, if anything, it licenses at the bedside in the meantime.

### 6.6 What the framework permits at the bedside today

The answer is limited, and one negative rule takes precedence over every positive inference the framework suggests. Predicted hepatic non-response is not by itself sufficient grounds for withholding or discontinuing a GLP-1 receptor agonist. The reason does not depend on the subgroup analysis and its power. The intention-to-treat cardiovascular benefit in SELECT was a 20% reduction in major adverse cardiovascular events. It was established in a population defined by established cardiovascular disease and a body mass index of 27 or above without diabetes. It was never conditioned on hepatic status at all, since the trial required no diagnosis of MASLD (74). Withholding the drug on the basis of predicted hepatic non-response therefore forfeits a benefit that was never contingent on hepatic response in the first place. The extrapolation should be stated plainly: SELECT was a secondary-prevention population, and the rule is on firmest ground there and weakest in primary prevention.

The rule extends to two behaviors that occur more often than outright withholding. The first is discontinuation during treatment when a non-invasive hepatic test fails to improve; the second is a payer conditioning continued coverage on demonstrated hepatic response. Both apply a hepatic criterion to a drug whose best-established benefit in this population is not hepatic. None of this displaces the legitimate reasons to stop, which are intolerance, absence of weight response, cost, and patient preference.

It follows that what this framework changes in first-line drug selection today is nothing, and the constraint producing that answer is worth stating exactly. Wherever cardiovascular risk is present, the incretin carries outcome evidence no comparator can match, and no predicted hepatic probability outweighs evidence of that class. The drug-selection module is therefore inert in precisely the population in which MASH is usually treated. The scenario in which it would first become live is correspondingly narrow, and specifying it is more useful than claiming broader reach. It requires a patient in whom the cardiovascular argument is absent or already satisfied, an operative hepatic endpoint, and two agents between which the evidence genuinely differs. A patient without established cardiovascular disease, with fibrosis as the treatment indication, for whom an incretin and a thyroid hormone receptor-β agonist are both reasonable, is the closest current approximation. Even there, the framework cannot decide. The only input bearing on the choice is the diabetes signal, and that sits in tier 2 on across-trial subgroup comparison without formal interaction testing (76–78). What would make it live is not a new marker but formal interaction testing on individual participant data from existing trials, which requires no new recruitment. That is the specific gate between the present framework and a decision it could support. Until it is passed, the immediate value of the framework is in study design. Completed trials with stored biospecimens can be interrogated now, and trials beginning now can prespecify the stratification that retrospective analysis can only approximate.

Both the negative rule and the narrowness of the drug-selection scenario rest on one premise: that the cardiovascular benefit of these agents does not follow the hepatic axis. That premise has so far been asserted rather than examined. It is examined next, because the dominant cause of death in this population is cardiovascular, and a framework calibrated only for the liver would be answering the lesser question.

## 7 Endpoint-specific calibration: why hepatic stratification does not extend to cardiovascular benefit

Any framework that stratifies MASLD patients for therapy must answer to an outcome outside the liver. Cardiovascular disease, and not hepatic decompensation, is the dominant cause of death in this population. The question this raises is uncomfortable. Stratification variables selected to predict hepatic response need not predict cardiovascular benefit. If the two axes dissociate, a framework optimized for the liver could be uninformative or actively misleading about the outcome that matters most. Three observations bear on it, drawn from different levels of evidence and resting on different assumptions, and they converge. At the level of the randomized trial, cardiovascular benefit in SELECT does not track baseline fibrosis risk. At the level of the single variant, HSD17B13 rs72613567 protects against steatohepatitis and cirrhosis without protecting against steatosis. A hepatic exposure and outcome therefore already come apart under genetic control (37). At the level of the clinical phenotype, lean MASLD combines a low probability of adiposity-driven hepatic response with cardiovascular risk that is at least comparable with non-lean disease. Genetic instrumental-variable data corroborate the same separation but rest on assumptions weaker than any of the three. They are treated below as supporting rather than load-bearing (**Figure 4**). What this section settles is not a question about comorbidity but a question about the framework’s output: whether one calibration can serve both endpoints, or two are required.

**Figure 4:**
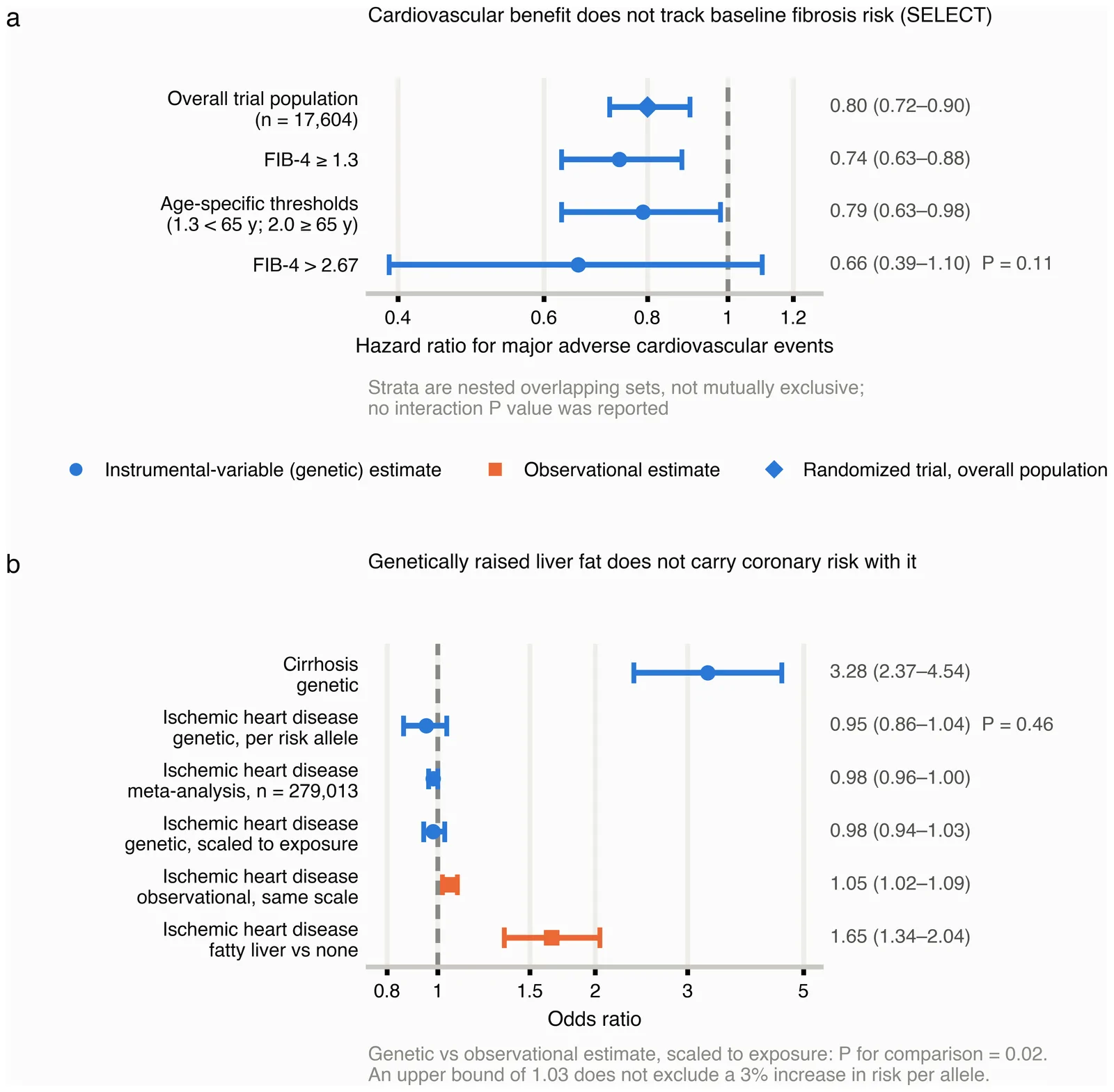
Hepatic and cardiovascular endpoints come apart, which is why a single predicted probability cannot serve both. **(a)** Cardiovascular outcomes in SELECT by baseline FIB-4 stratum. The strata are nested overlapping sets rather than mutually exclusive groups, and no interaction P value was reported. **(b)** Effect of the PNPLA3 I148M variant on hepatic and cardiovascular endpoints, comparing instrumental-variable with observational estimates. The two panels are plotted on separate x axes and are not directly comparable. **(a)** reports hazard ratios from a randomized trial, and **(b)** reports odds ratios from instrumental-variable and observational analyses. Symbol shape encodes the type of estimate: a diamond for the randomized trial overall, a circle for an instrumental-variable estimate, a square for an observational estimate. Estimate type is therefore identifiable without reliance on color. An upper confidence bound of 1.03 does not exclude a 3% increase in risk per allele.

### 7.1 Cardiovascular benefit does not track baseline FIB-4 category

Cardiovascular benefit from semaglutide is established, and no effect modification by baseline fibrosis risk has been detected. The highest-risk stratum was nonetheless underpowered to exclude it. In SELECT, 17,604 patients with preexisting cardiovascular disease and overweight or obesity but without diabetes were randomized to once-weekly semaglutide 2.4 mg or placebo. The primary composite was cardiovascular death, non-fatal myocardial infarction, or non-fatal stroke. It occurred in 6.5% versus 8.0% of patients over a mean follow-up of 39.8 months (hazard ratio 0.80, 95% CI 0.72–0.90) (74). A prespecified secondary analysis examined participants at high risk of substantial liver fibrosis by baseline Fibrosis-4 (FIB-4) score. Cardiovascular events were reduced by 26% in those with a score of 1.3 or above (HR 0.74, 95% CI 0.63–0.88). Age-specific thresholds of 1.3 below age 65 and 2.0 at age 65 or above gave a 21% reduction (HR 0.79, 95% CI 0.63–0.98). In the small group with a score above 2.67, the reduction was 34%, and the estimate did not reach significance (HR 0.66, 95% CI 0.39–1.10, P = 0.11) (75).

Three features constrain how that analysis can be read. First, the groups are not mutually exclusive strata but nested and overlapping sets. Patients scoring above 2.67 are contained within those scoring 1.3 or above, and the age-specific definition is a different cut of the same continuous variable. No interaction p value was reported. The ordering of the three hazard ratios therefore cannot be read as a gradient across fibrosis risk, in either direction. Second, Fibrosis-4 includes an age term. This population was aged predominantly above 45, with established cardiovascular disease and substantial background medication. A high score there identifies a group differing from a low-score group in more than hepatic fibrosis. Third, SELECT neither required a diagnosis of MASLD nor performed elastography or biopsy. The hepatic phenotype of these subgroups is therefore inferred from a biochemical index rather than measured. The reported 28% greater reduction in fatty liver index is likewise computed in part from body mass index and waist circumference. It is not independent hepatic evidence under a weight-loss agent. What survives is the finding that matters here. The reductions are not ordered by fibrosis risk. The data are best read as benefit consistent with the overall trial population rather than amplified by hepatic risk, which is how the authors themselves frame it. For the framework, the consequence is specific: the cardiovascular arm cannot be calibrated on hepatic risk, because hepatic risk does not order the benefit.

### 7.2 Why the genetic axis cannot be carried across to cardiovascular risk

Instrumental-variable data point the same way, on assumptions weaker than those of the trial-level and variant-level observations above. Liver fat is associated with ischemic heart disease observationally, with an odds ratio of 1.65 (95% CI 1.34–2.04) in individuals with versus without fatty liver disease (79). Mendelian randomization using PNPLA3 I148M as an instrument does not reproduce it. The variant behaved as expected on the hepatic side, conferring an odds ratio of 3.28 (95% CI 2.37–4.54) for cirrhosis. Across 279,013 individuals, and scaled to the exposure, the odds ratio for ischemic heart disease per M-allele higher genetically determined liver fat was 0.98 (95% CI 0.94–1.03). The observational estimate was 1.05 (95% CI 1.02–1.09), and a formal comparison of the two yielded P = 0.02 (79). The authors concluded that lifelong, genetically determined liver fat is not causally associated with coronary risk and that the observational association reflects confounding or reverse causation. The relevance here is narrow but direct. The variant anchoring the genetic axis of this framework is the one this analysis finds uninformative about coronary risk.

Two features of the design bound how far that conclusion travels, and **Supplementary Note 5** sets them out. The more consequential is that the exposure is pathway-specific. PNPLA3 I148M raises hepatic fat by retaining lipid within the droplet rather than by altering export, leaving VLDL production and clearance indistinguishable between genotypes (80). The instrument therefore captures a form of hepatic fat that leaves the atherogenic lipoprotein axis untouched. That is not the exposure seen in observational cohorts, where steatosis arrives with insulin resistance and atherogenic dyslipidemia. A null result for the first tells us little about the second. The confidence interval is a further caution: an upper bound of 1.03 does not exclude a 3% increase in risk per allele.

One thing the analysis does establish needs no assumption beyond the validity of the instrument itself. The same genetic exposure that raises cirrhosis risk more than threefold leaves coronary risk unmoved. No fibrosis-specific instrument is needed to make that point, and none was used here; this analysis speaks to hepatic fat alone. The consequence for stratification is direct. The genetic axis of the framework is built on variants that govern hepatic lipid handling. It carries information about hepatic outcomes that should not be assumed to transfer to cardiovascular ones. A genetic risk score calibrated for fibrosis progression should not be used to rank cardiovascular risk. Whether hepatic fibrosis contributes independently to cardiovascular events is a separate question that these data do not address, and it belongs on the list of what is missing.

### 7.3 Implications for endpoint-specific calibration

The lean phenotype is where the two axes separate most visibly, and it clarifies what form the framework’s output must take. Lean MASLD carries cardiovascular risk that is at minimum comparable with, and by some analyses greater than, that of non-lean disease, despite the lower body mass index. The divergence in the mortality data remains unresolved. Lean patients also carry distinctive metabolic, genetic, and microbial profiles alongside greater visceral adiposity and sarcopenia (81). A patient can thus sit low on an adiposity-driven treatment-response axis while sitting high on cardiovascular risk, and no single stratification output can serve both readings. This is the third of the three observations, and the one requiring no genetic assumption at all. Taken with the absence of a fibrosis-risk gradient in SELECT, and with the steatosis-versus-steatohepatitis dissociation at HSD17B13, it supports an endpoint-specific framework. Such a framework produces separate calibrations for predicted hepatic response and for cardiovascular risk reduction rather than a single composite score. The case does not rest on the Mendelian randomization result; were that analysis set aside entirely, the trial-level and phenotype-level observations would still require the separation.

That cardiovascular benefit does not track the FIB-4 category also tempers the cost of getting the hepatic axis wrong. A patient misclassified as a likely hepatic non-responder still stands to gain cardiovascular protection from a GLP-1 receptor agonist. That argues against using predicted hepatic non-response as a reason to withhold the drug. It also means that the drug-selection question raised by the contested diabetes signal is currently unanswerable on cardiovascular grounds. No comparison of incretin against thyroid hormone receptor-β agonism has used cardiovascular events as a primary endpoint (76).

What is established is that GLP-1 receptor agonists reduce cardiovascular events substantially and consistently across baseline FIB-4 categories. It is also established that the causal link from hepatic fat to coronary disease is weaker than observational data suggest. What is missing leaves the cardiovascular arm of the framework wholly untested and is set out with the corresponding designs in **Table 5**. One question outranks the others for a clinician who must make a single prescribing decision: whether any pretreatment variable predicts hepatic and cardiovascular benefit concordantly.

## 8 Conclusion and outlook

Response to GLP-1–based therapy in MASLD/MASH is heterogeneous in a way that host genotype, gut microbial features, and clinical phenotype together help explain. The contribution of this review lies not in new evidence but in two organizing decisions about evidence already available. Existing reviews treat the mechanism of incretin action, genetic susceptibility, and the gut–liver axis as separate literatures, each internally coherent and each insufficient alone for prediction. Assembling them required sorting the constituent findings by maturity rather than by topic. Secure results carry weight in a stratification decision, whereas contested and extrapolated ones generate hypotheses instead. It also required separating hepatic from cardiovascular calibration. Three independent observations support that separation, and Section 7 sets them out.

The limitations of the resulting framework are substantial and specific. Both of its pretreatment pillars rest on extrapolation. The genotype dependence of hepatic benefit is established for dietary weight reduction and in genotype-defined experimental systems, not for incretin therapy. The predictive value of baseline microbiota is established for a dietary prebiotic rather than for a GLP-1 receptor agonist. No prospective trial has stratified enrollment by genotype or by baseline microbial profile. The cardiovascular arm is wholly untested, since no stratified trial has used cardiovascular events as a primary endpoint. The one axis that is routinely measurable is itself unsettled, and the evidence that diabetes status modifies drug-specific fibrosis response derives from across-trial subgroup comparison without formal interaction testing. A further asymmetry deserves statement. The genetic axis is invoked on the ground that risk-allele carriers extract more hepatic benefit per kilogram lost. Yet, the same allele is associated with losing less weight, and no study has reported the net effect. Section 6.5 specifies the mediation decomposition that would resolve it from existing trial data. The proposed ordering of the axes by evidence maturity is a working hypothesis rather than an empirically derived weighting.

What follows from this is an agenda rather than an instrument. The analyses that would move the framework forward are ordered by what they require. Three need no new recruitment. They are formal interaction testing on individual participant data, nested modeling of genotype against microbial features, and the mediation decomposition of the genetic axis. Those needing stored biospecimens come next, since completed incretin trials can be interrogated for genotype-stratified and microbiota-stratified response without enrolling a patient. Only then does prospective stratification become worth its cost, and the standardization of sampling and sequencing must precede rather than accompany it. Across all of these, external validation must span ancestries. A framework calibrated in one population and applied in another would compound existing disparities in liver disease outcomes rather than reduce them.

The obstacles beyond that are correspondingly specific. Experimental systems must render genotype and microbiota manipulable together, since neither domain in isolation reproduces their interaction. Integration must contend with axes on different timescales, a genome fixed for life against a community that shifts under treatment. Any model fusing these inputs must be inspectable, because a weighting that cannot be examined will not be trusted for a prescribing decision. Stratified trials will reorder the weighting proposed here and may refute the transfer of the genotype effect to incretin therapy altogether. Either outcome would advance the field further than continuing to treat these determinants in isolation. In the meantime, one rule holds regardless of how the framework develops: predicted hepatic non-response is not by itself sufficient grounds for withholding or discontinuing a GLP-1 receptor agonist.
