# Supplementary material for: Predicting response to GLP-1–based therapy in MASLD/MASH: an evidence-tiered, endpoint-specific framework built on host genotype and gut microbial features
Source: Front Endocrinol (Lausanne). 2026 Oct 27;17:1962068. doi: 10.3389/fendo.2026.1962068 (PMC13749324; doi:10.3389/fendo.2026.1962068)
Supplement: Supplementary file 1 [file SupplementaryFile1.docx]

# Supplementary Material

**Predicting Response to GLP-1–Based Therapy in MASLD/MASH: An Evidence-Tiered, Endpoint-Specific Framework Built on Host Genotype and Gut Microbial Features**

Guojian Zhu, Yucao Huang, Yun Huang, Wenlong Yang

## Supplementary Note 1. Disaggregating medication-driven microbial shifts from disease and response signatures

Referenced from Section 6.2 of the main text.

Most patients with MASH receive several drugs that alter the gut community. Metformin is the clearest case: it shifts composition in treatment-naive type 2 diabetes, and part of its glycemic effect is attributable to that shift (47). It also produces weight loss accompanied by microbial change in non-diabetic individuals (48). SGLT2 inhibitors, proton pump inhibitors, statins, and recent antibiotic exposure alter composition or bile acid handling in the same population. A pre-treatment microbial profile measured in such a patient is therefore a composite of disease, drug, and diet.

The response-predictive component of that composite cannot be recovered by covariate adjustment, and the reason is structural rather than a matter of insufficient sample size. Adjusting for medication exposure as a binary term treats a mediated pathway as though it were a confounder, and removes part of the signal along with the noise. Adjusting instead for the microbial features that the medication alters conditions on an intermediate variable, which induces bias of its own. Neither specification estimates the quantity of interest.

Three approaches avoid the problem rather than model it, and they differ in what they can identify.

**Stratified derivation.** Deriving the predictor separately within medication strata tests whether the same features carry signal in each. Features that predict response only in the unexposed stratum are medication artifacts; features that predict in both are candidates for the axis. This costs power and requires adequate representation of unexposed patients, but it requires no new design and can be applied to existing collections. Stratum-specific reporting should be an expectation of any such analysis rather than a sensitivity check appended to it.

**Within-patient change.** A medication-driven microbial state is largely stable while that treatment continues. The component of the baseline profile that shifts under incretin therapy is therefore less contaminated by concomitant medication than the absolute baseline is. This approach trades the pre-treatment property that makes the axis clinically attractive for a cleaner signal, so it serves as a validation strategy rather than as a deployable assay.

**Prespecified regimen stratification.** Nesting the microbial substudy in patients on a stable regimen, with regimen recorded as a prespecified stratification variable at enrollment rather than as a post hoc covariate, is the only one of the three that identifies the disease-response signature cleanly. It is also the only one that must be specified before the trial begins. Trials that stored stool without recording concomitant medication in this form cannot support the analysis retrospectively.

This constraint is the most concrete reason the microbial axis remains in Tier 2 and Tier 3, rather than a reflection of insufficient sequencing effort. Excluding all exposed patients would remove much of the population the framework is meant to serve, since coexisting type 2 diabetes is both an inclusion criterion for the clinical axis and the main reason for metformin use.

## Supplementary Note 2. Mediation analysis of the genotype–weight-loss–hepatic-benefit trade-off

Referenced from Section 6.5 of the main text.

The genetic axis is invoked on the ground that PNPLA3 risk-allele carriers extract a steeper reduction in liver stiffness per kilogram lost, yet the same allele is associated with losing less weight (10). The two effects run in opposite directions and no study has reported their net. Counterfactual mediation analysis supplies the form in which that net can be estimated, with genotype as the exposure, weight lost as the mediator, and hepatic improvement as the outcome.

**Decomposition.** The total effect of genotype separates into a natural indirect effect transmitted through weight loss and a natural direct effect capturing the residual hepatic return per kilogram lost. Carriers reaching the 10% weight-loss threshold less often should appear as an indirect effect adverse to hepatic benefit. A steeper hepatic return per kilogram should appear as a favourable direct effect. The quantity that decides whether the axis is worth measuring is the total effect, which is their sum and may be null while both components are large. Reporting either component alone would misrepresent the axis in a predictable direction.

**Exposure–mediator interaction.** Interaction between genotype and weight lost must be permitted in the model rather than assumed absent, because the claim under test is precisely that genotype alters the slope relating weight loss to hepatic change. A model constraining that interaction to zero cannot estimate the effect the framework proposes.

**Identifying assumptions.** Four conditions identify the decomposition: no unmeasured exposure–outcome confounding, no unmeasured exposure–mediator confounding, no unmeasured mediator–outcome confounding, and no mediator–outcome confounder affected by the exposure. Genotype’s randomization at conception secures the first two. The third is the vulnerable one here, because adiposity, diet, and glucose-lowering co-treatment all plausibly confound the path from weight lost to hepatic improvement, and none is fully measured in the trials that would supply the data. Sensitivity analysis across a range of unmeasured mediator–outcome confounding is therefore obligatory rather than optional, and the reported estimate should be accompanied by the magnitude of confounding that would explain it away.

**Feasibility.** The analysis requires no new recruitment. Randomized incretin trials with stored DNA, serial weight measurements, and paired biopsies contain everything it needs. Reporting the decomposition would convert the net trade-off from a stated uncertainty into an estimated quantity with a confidence interval, and would determine whether the genetic axis survives as an efficiency modifier or should be removed from the framework.

## Supplementary Note 3. Fecal versus mucosal sampling, and a nested validation design

Referenced from Section 5.3 of the main text.

Stool samples the distal colonic lumen. The interactions on which the microbial axis depends are not located there. Intestinal FXR activation and bile acid reabsorption occur in the terminal ileum (26,27), and the mucosa-adherent community that contacts the epithelium is not the community a fecal sample captures. A fecal profile is therefore a surrogate for the compartment of interest rather than a measurement of it.

Sampling that compartment directly requires endoscopy or a capsule device. Neither is defensible as a routine pre-treatment test for a drug that is otherwise prescribed on clinical grounds alone, and neither scales to the population in which the framework would operate. The constraint is practical rather than conceptual, but it is binding.

Two consequences follow, and they point in opposite directions.

The first is a limit on interpretation. A fecal marker cannot be assumed to report the mucosal process it is invoked to represent. A fecal predictor that failed external validation would therefore leave the underlying mechanism untested rather than refuted, and a fecal predictor that succeeded would not establish that the mucosal interaction is the operative one.

The second is a reason the assay may nonetheless work. A predictive marker does not require mechanistic fidelity. It needs only to share an upstream cause with the response mechanism, which is the marker-versus-lever distinction drawn at the end of Section 5. The resistant-starch model was built on stool and externally validated on stool, reaching an area under the curve of 0.74 (95% CI 0.66 to 0.81) in an independent multicenter cohort (12). A distal luminal specimen can therefore carry response information about a process that is not located in the distal lumen.

**Nested validation design.** What this argues for is a validation strategy rather than abandonment of the specimen. Fecal sampling should serve as the deployable assay, with paired mucosal sampling nested in a subset of participants who are undergoing endoscopy for an independent clinical indication. The nested comparison would establish how far the fecal signal tracks the ileal one, and would indicate whether a fecal predictor that fails validation has failed as a marker or as a proxy. Until that comparison exists, a fecal predictor should be treated as an empirical marker under test rather than as a measurement of the mucosal interaction the mechanism describes.

## Supplementary Note 4. Ordering of candidate microbial assays by proximity to function

Referenced from Section 6.4 of the main text.

The microbial axis is specified functionally rather than taxonomically, because every microbial finding assembled in the main text is taxonomic while the mechanism the axis invokes is metabolic. The candidate assays order themselves by how closely each approaches microbial activity rather than microbial identity.

**Gene-level targets.** Bile salt hydrolase, the *bai* operon that generates secondary bile acids, the butyrate kinase and butyryl-CoA transferase genes, and polysaccharide utilization loci are the most accessible targets, and can be quantified from the same sequencing data that yield taxonomic composition. Their limitation is fundamental rather than technical: the presence of a gene establishes neither its expression nor the metabolic flux through it, and communities differing in gene content may not differ in output.

**Metatranscriptomics.** Sequencing community RNA addresses expression directly and removes the first of those two gaps. It has no current clinical feasibility, being sensitive to sample handling in ways that a routine pre-treatment assay could not accommodate, and it still does not measure flux.

**Targeted metabolomics.** Quantification of fecal and serum bile acids, short-chain fatty acids, and tryptophan-derived indoles sits closest to function while remaining the most tractable to clinical implementation. It measures the output of the community rather than its capacity, which is the quantity the mechanism implicates.

**Absolute quantification at strain or clade level.** This would resolve ambiguities that genus-level reporting cannot, and it bears directly on the *Prevotella* discrepancy set out in Section 5.3, where clades differing by more than a tenth of their genomes are reported under a single genus name.

This ordering rests on more than the convenience of measurement. The differential responsiveness of bile acid metabolism relative to community-level indices, documented in the two randomized trials of incretin therapy discussed in Section 3, is itself an argument for assaying metabolic output rather than membership.

## Supplementary Note 5. Design limitations of the Mendelian randomization evidence on liver fat and coronary risk

Referenced from Section 7.2 of the main text.

The instrumental-variable analysis that finds no causal association between genetically determined liver fat and ischemic heart disease (79) is treated in the main text as supporting rather than load-bearing evidence. Two features of its design account for that placement.

**Single-instrument pleiotropy cannot be diagnosed.** The analysis uses PNPLA3 I148M as its sole instrument. The standard sensitivity methods for horizontal pleiotropy (MR-Egger, weighted median, and MR-PRESSO) all require multiple instruments and therefore cannot be applied. Instrument strength was not reported, so weak-instrument bias cannot be excluded either. A single-instrument analysis rests on the exclusion restriction being true rather than on evidence that it is.

**The exposure is pathway-specific.** This is the more consequential limitation. PNPLA3 I148M raises hepatic fat through retention of lipid within the droplet rather than through altered hepatic export. In nine homozygotes for 148M compared with nine wild-type controls, liver fat was more than three-fold higher, while production rates of apolipoprotein B100 and triglyceride in both VLDL1 and VLDL2, and the fractional clearance rates of each, were all statistically indistinguishable between genotypes (80).

The instrument therefore captures a form of hepatic fat that leaves the atherogenic lipoprotein axis untouched. That is not the same exposure as the hepatic fat observed in cohort studies, where steatosis arrives together with insulin resistance and atherogenic dyslipidemia. A null causal estimate for the first tells us little about the second, and the two should not be treated as estimates of the same quantity.

**Precision.** The confidence interval is a further caution. An upper bound of 1.03 does not exclude a 3% increase in risk per allele, so the analysis is compatible with a small adverse effect as well as with none.

None of these limitations bears on the observation the main text draws from this analysis, which requires no assumption beyond instrument validity.

## Supplementary Note 6. Oligogenic risk scores and the calibration problem in ancestrally diverse populations

Referenced from Section 6.4 of the main text.

The genetic axis of the framework is operationalized as an oligogenic risk score summing PNPLA3 and TM6SF2 risk alleles and subtracting the protective HSD17B13 genotype (70). Two properties of that construction bear on how it must be validated before use.

**The subtraction is biological, not arithmetic.** The protective allele is not merely a negative weight in a sum. In one human dataset the HSD17B13 splice variant mitigated the liver injury associated with the PNPLA3 risk allele (37), so the score encodes an interaction between loci rather than an independent additive contribution from each. A score fitted without allowance for that interaction may misestimate risk in carriers of both.

**Calibration, not discrimination, is the portability problem.** These are oligogenic scores built on a handful of large-effect loci, not genome-wide polygenic scores aggregating thousands of small effects. The two fail differently across ancestries. Genome-wide scores lose discrimination when transported, because linkage disequilibrium patterns differ and tag variants no longer track causal ones. An oligogenic score built on directly genotyped causal variants largely retains its discrimination, because the variants themselves carry the effect. What it loses is calibration: allele frequencies differ markedly between ancestral populations, and the PNPLA3 risk allele is most frequent in Hispanic populations and least frequent in individuals of African ancestry (32). The absolute risk attached to a given percentile of the score therefore shifts between populations even when the ranking within each remains valid.

The operational consequence is that a percentile threshold established in one population cannot be carried into another without recalibration. A framework deployed on uncalibrated thresholds would misclassify systematically by ancestry, and would compound existing disparities in liver disease outcomes rather than reduce them. This is why external validation across ancestries is listed in Section 6.5 as a requirement for Tier 1 evidence, secure as that tier otherwise is.

**No incremental discrimination has been reported.** For any such score in this disease, the quantitative gain over clinical variables alone has not been estimated (9). This is a gap in the published literature rather than an omission from the present framework, and it is one of the analyses that existing cohorts could supply without new recruitment.

## Supplementary Note 7. The human interventional record for microbiome-targeted therapy in MASLD

Referenced from Section 5.4 of the main text.

The main text summarizes the shape of this record. It is set out here in full, because the detail of each trial’s design is what licenses the conclusion drawn, and because a reader assessing the microbial axis should be able to see the negative evidence at the same resolution as the positive.

**The largest trial.** A randomized controlled trial assigned 104 patients to a synbiotic or placebo for 10 to 14 months. Liver fat measured by magnetic resonance spectroscopy and two validated fibrosis scores were co-primary endpoints, so the trial was powered and prespecified for the outcomes that matter. The intervention altered the fecal microbiome and moved none of the three (56).

Two findings within it bear directly on the argument of this review. First, change in microbiota composition was associated with neither liver fat nor fibrosis markers, so no dose-response relationship linked microbial change to hepatic outcome; the intervention did what it was designed to do to the community without that translating into hepatic benefit. Second, in fully adjusted models weight change was the only variable independently associated with a decrease in liver fat, which places this trial alongside the incretin evidence in Section 2.2 rather than in tension with it.

**Smaller probiotic studies.** A multispecies probiotic reduced hepatic fat modestly, but the difference from placebo did not survive adjustment for weight change (57). Pasteurized *Akkermansia muciniphila*, a non-viable preparation, improved insulin sensitivity by 28.6% in a 32-participant proof-of-concept trial that had no prespecified hepatic endpoint. It did so without altering community structure (58), which cuts against the narrative this study is usually cited to support: an intervention that improves metabolic parameters without changing the community is evidence against the community being the operative mechanism, not for it.

**Fecal microbiota transplantation.** Three randomized trials reported no improvement in hepatic fat or insulin sensitivity. One was double-blind and delivered three sequential transplants, with the between-group difference in magnetic resonance proton density fat fraction reaching a p value of 0.50. Another used paired biopsies, and histological change amounted only to a trend (59-61). The single positive transplantation trial differed from these in three respects that bear on its weight: it was open-label, it compared transplantation against oral probiotics rather than against placebo, and it followed patients for one month (62). The contrast between the blinded negative trials and the unblinded positive one is itself informative.

**Meta-analysis.** Across twenty-one trials, probiotics and synbiotics lowered alanine aminotransferase by 11.23 IU/L and liver stiffness by 0.70 kPa. Heterogeneity was 90.6% and 93.4% respectively, which is high enough that the pooled estimates should not be read as describing a consistent effect. The authors noted that no trial had then examined sequential liver biopsies (63).

**Histological endpoints since.** Only two microbiome-targeted trials have made liver histology their stated primary endpoint, enrolling 14 and 39 patients. In the larger, only 15 participants completed paired biopsy (64,65). Neither is adequately powered for a histological endpoint.

**What this record supports.** The field has no adequately powered histological test of microbial manipulation, and the one trial powered for quantitative hepatic endpoints was negative. This is the basis for the marker-versus-lever distinction drawn at the end of Section 5: baseline composition forecast response to a substrate delivered to the community, while deliberate manipulation of that composition failed to move hepatic endpoints in the trial designed to detect it. The two findings are compatible, and keeping them apart is what allows the microbial axis to be proposed as a marker without implying that it is a target.

## Supplementary Note 8. Causal mechanism of the PNPLA3 I148M variant

Referenced from Section 4.1 of the main text.

The main text treats the PNPLA3 risk allele as a substrate for therapy rather than only as a marker of risk. That treatment rests on the mechanism being established rather than inferred, which distinguishes this locus from the other three and is why it is the only one with an antisense oligonucleotide in clinical trials. The supporting experiments are set out here.

**The variant is sufficient to cause steatosis.** Knock-in mice carrying I148M accumulate the PNPLA3 protein on hepatic lipid droplets and develop hepatic steatosis (34). This establishes that the variant acts in the liver and that the phenotype follows from the variant itself rather than from a linked locus.

**The mechanism is not loss of function.** Ablation of wild-type PNPLA3 does not cause steatosis, so the phenotype cannot be explained by the variant simply failing to do what the wild-type protein does. A catalytically dead variant reproduces the phenotype, which excludes loss of catalytic activity as the operative mechanism (34). Together these two results point to a gain of function, in which the variant protein does something the wild-type does not.

**The gain of function is protein accumulation.** The variant protein accumulates on lipid droplets because it evades ubiquitylation and subsequent proteasomal degradation (35). Wild-type PNPLA3 is turned over; I148M is not, and the accumulated protein interferes with lipid mobilization from the droplet.

**Why this matters for therapy.** A gain-of-function mechanism driven by protein accumulation is addressable by reducing the amount of protein, which is what an antisense oligonucleotide or an RNA interference therapeutic does. A loss-of-function mechanism would not be. This is the basis for the genotype-restricted trials discussed in Section 4.3, and it is also why the same reasoning does not transfer automatically to TM6SF2 or MBOAT7, where the mechanism is less completely resolved.

## Supplementary Note 9. The resistant-starch trials: baseline microbiota as a predictor of response

Referenced from Section 5.3 of the main text.

These trials are the empirical basis for the claim that pre-treatment microbial features can forecast response to a metabolic intervention in MASLD. Because that claim carries most of the weight of the microbial axis, the evidence is set out here at full resolution.

**The first trial.** A four-month randomized trial of resistant starch reduced intrahepatic triglyceride content by 9.08% in absolute terms, and by 5.89% after adjustment for weight loss. The effect tracked changes in specific microbial species including *Bacteroides stercoris* (49). The adjustment matters: roughly a third of the hepatic effect was attributable to weight loss, leaving a component that was not.

**The second trial and its treatment of heterogeneity.** A subsequent randomized, placebo-controlled trial of resistant starch in MASLD addressed response heterogeneity directly rather than reporting a group mean. Roughly a third of participants derived limited benefit. Multi-omic analysis, together with transfer of patient microbiota into antibiotic-depleted mice, identified baseline microbiota as the dominant determinant of that heterogeneity (12).

**The mechanism isolated.** Population stratification implicated high *Prevotella* abundance, which impaired utilization of the intervention and marked low responders. The causal role of the genus was supported by co-culture experiments and by transfer into mice, not by stratification alone. This distinguishes the finding from the many microbiome associations that rest on stratification only, and it is why the result is treated as load-bearing in the main text.

**The predictive model and its two estimates.** A simplified model integrating baseline microbial and clinical features separated responders from non-responders. It reached an area under the curve of 0.87 in the trial in which it was built, and 0.74 (95% CI 0.66 to 0.81) in an independent multicenter validation cohort (12).

The gap between the two estimates should be read as informative rather than as a shortfall. Feature selection and synthetic oversampling were both performed within the derivation trial, which inflates the internal estimate by construction. The external figure of 0.74 is therefore the one that should be cited, and the main text cites only that one. Reported discrimination in the 0.9 range elsewhere in this literature, as noted in Section 5.2, typically derives from derivation cohorts without external validation and should be read the same way.

**What it does and does not establish.** It establishes that baseline microbial composition can carry response information for an intervention delivered to the community. It does not establish that the same holds for a drug acting on host receptors, and Section 5.3 sets out why that transfer is not a short step. The structural difference between the two causal architectures is the reason the microbial axis sits in Tier 3 for incretin response despite this trial being, on its own terms, a strong result.

## Supplementary Note 10. Randomized trials of incretin therapy examining the gut microbiome directly

Referenced from Section 3 of the main text.

Two randomized trials have examined what incretin therapy does to the gut community in patients. Both were conducted in type 2 diabetes with glycemic rather than hepatic endpoints, which is why the main text treats them as bounding the mechanism rather than establishing it.

**Liraglutide and sitagliptin, 12 weeks.** A randomized placebo-controlled trial in adults with type 2 diabetes found that liraglutide altered fecal secondary bile acids. It affected neither alpha nor beta diversity, and no compositional change tracked clinical response (30). The title of that report states that the drugs had no effect on intestinal microbiota composition.

**Liraglutide added to metformin, youth-onset diabetes.** A randomized trial in youth-onset type 2 diabetes did detect taxon-level shifts with liraglutide added to metformin. Bile acid changes there correlated with improved glycemia; the microbial shifts themselves did not (31).

**What the pair supports, and what it does not.** Read together, the two trials show that bile acid metabolism responds to incretin therapy more reliably than community-level indices do. That asymmetry is the empirical basis for two positions taken in the main text: that the microbiome is a modifier of the gut–liver relay rather than its principal effector (Section 3), and that assays approaching metabolic output should be preferred to assays of community membership (Section 6.4, Supplementary Note 4).

Three limits should be kept in view. Both trials used glycemic endpoints, so neither speaks directly to hepatic outcome. Unchanged alpha and beta diversity are not evidence of unchanged composition, since diversity indices are insensitive to compositional turnover that preserves overall structure. And the populations were diabetic, with metformin co-exposure in the second trial, which is the confounding discussed in Supplementary Note 1.

Neither trial was designed to test whether baseline microbiota predicts response, which remains untested for any GLP-1 receptor agonist.

Reference numbering follows the main text.
